# Patterns in health care use and intensity for diagnosed and undiagnosed cognitive impairment in the older australian community: Implications for primary care management

**DOI:** 10.1016/j.ssmph.2024.101693

**Published:** 2024-07-02

**Authors:** Anam Bilgrami, Mona Aghdaee, Yuanyuan Gu, Henry Cutler, Katya Numbers, Nicole A. Kochan, Perminder S. Sachdev, Henry Brodaty

**Affiliations:** a Macquarie University Centre for the Health Economy, Macquarie Business School & Australian Institute of Health Innovation, Macquarie University, Macquarie Park, NSW, 2113, Australia; b Centre for Healthy Brain Ageing (CHeBA), Discipline of Psychiatry & Mental Health, University of New South Wales, Sydney, NSW, 2052, Australia; c Neuropsychiatric Institute, The Prince of Wales Hospital, Sydney, NSW, 2031, Australia

**Keywords:** Cognitive impairment, Dementia, Health care, Costs, Primary care, Service gaps

## Abstract

**Objectives:**

While the economic burden imposed by dementia is well-documented, findings are mixed on health care use for those with mild cognitive impairment (MCI). Our objective was to analyse annual, non-hospital medical and pharmaceutical use patterns for older people with undiagnosed MCI and diagnosed dementia, living in the Australian community.

**Methods:**

We analysed panel data from a community sample, the Sydney Memory and Ageing Study (Australia), linked to administrative data on health care use, using two-part models to estimate the probability of using health care and the annual costs incurred by study participants.

**Results:**

People with MCI, unaware of their diagnoses, were significantly less likely to incur annual pathology and diagnostic imaging costs relative to cognitively normal individuals. This effect was concentrated in individuals with MCI who had non-amnestic symptoms, lived alone, or had limited carer support. Compared to cognitively normal individuals, people with MCI were predicted to have slightly lower annual costs for broad medical care categories related to the management and diagnosis of cognitive impairment, and people with dementia, substantially higher professional attendances, and pharmaceutical costs. These findings were consistent across estimation models adjusting for attrition over the study.

**Policy implications:**

Diagnosis and symptom management in primary care may enable individuals with MCI to improve their quality of life and prevent more costly future health care use. However, our study found potential gaps in medical service use for people with undiagnosed MCI in the community, especially when they had less support or did not have memory symptoms. Primary care services may need to better diagnose and target these individuals.

## Highlights

• Evidence is mixed on health care use patterns for mild cognitive impairment.
• Timely diagnosis may improve quality of life for mildly cognitively impaired people.
• People with mild cognitive impairment, unaware of their mild cognitive impairment, are less likely to use medical care.
• This pattern is prevalent for people living alone and without carer support.
• Primary care services may need to better diagnose and target these individuals.

## 1 BACKGROUND

1

With continued demographic ageing, older adults with dementia and mild cognitive impairment (MCI), the intermediate stage between normal aging and early dementia (Brodaty et al., 2017), represent a growing population (Sachdev et al., 2010). This will bear on future patterns of health care service need, use, diagnoses, and management in ageing countries.

People with MCI have substantially higher rates of progression to dementia than the general population (Brodaty et al., 2013) and are often undiagnosed (Caspi et al., 2009). Communication of diagnoses, follow-ups and symptom management may enable individuals with MCI to improve their quality of life or make lifestyle changes to modify risk factors. Research is also ongoing on whether early intervention may slow progression to dementia (Dooley et al., 2020).

However, while MCI is highly prevalent in community samples (Brodaty et al., 2013), the diagnosis itself is unstable and heterogenous, which may account for variations in whether and how diagnostic information is communicated to patients by doctors (Carreras et al., 2021). While guidelines for the management of MCI in primary care exist (Woodward et al., 2022), little is empirically known about the current state of practice amongst older people with undiagnosed MCI in the community.

Past studies consistently find diagnosed dementia to be costly for health care systems (Cantarero-Prieto et al., 2020) but findings are mixed on health care use for those with diagnosed and undiagnosed MCI and only one study (limited to general practitioner visits) has been conducted in the Australian context (Jenkins et al., 2016). Some studies have found more cognitive impairment, across the spectrum, to be associated with increased health care utilisation and costs (Aigbogun et al., 2017; Fowler et al., 2012; Jenkins et al., 2016; Jönsson et al., 1999; Lenox-Smith et al., 2018; Wolstenholme et al., 2002; Zhu et al., 2013). Others have found no association between MCI and health care costs (Luppa et al., 2008; Roelands et al., 2003; Ton et al., 2017), including the use of primary care (Leibson et al., 2015).

While a recent US study finds medical costs to be higher for people with MCI than cognitively normal individuals (Long et al., 2022), several past studies report *lower* medical care use amongst undiagnosed people with low cognition living in the community and suggest that this population may be under-served in terms of accessing outpatient services [ (Caspi et al., 2009), (Callahan et al., 1995), (Ton et al., 2017), (Walsh et al., 2003)]. Similar studies on potential outpatient service gaps have not been conducted for the Australian population, which has substantially lower cost and time barriers to accessing healthcare than the US (Wimo et al., 2023).

Findings may be mixed across studies due to different measures of cognitive impairment, diagnostic criteria for MCI and populations (i.e., clinical versus community), and whether and how mild diagnoses are communicated. Past studies also suffer from several methodological limitations, including most employing cross-sectional [ (Caspi et al., 2009), (Jönsson et al., 1999), (Aigbogun et al., 2017), (Ton et al., 2017), (Luppa et al., 2008), (Walsh et al., 2003), (Leibson et al., 1999)] rather than longitudinal designs (Jenkins et al., 2016), costing based on self-reported service use which is likely to be biased for people with memory issues [ (Caspi et al., 2009), (Jönsson et al., 1999), (Aigbogun et al., 2017), (Luppa et al., 2008), (Walsh et al., 2003)], unreliable measures of cognitive function (Aigbogun et al., 2017) and not employing two-part models, which are needed to account for the particularities of health care cost distributions (Walsh et al., 2003). While some recent studies have explored the association between subjective wellbeing and health care use by older Australians including mediational pathways such as physical activity and diet [ (Kesavayuth et al., 2021), (Kesavayuth et al., 2023)], research is limited on mediators and moderators of health care use within the specific context of cognitive impairment.

In this study, we analyse non-hospital medical and pharmaceutical use and costs associated with cognitive impairment in the older Australian community. Our sample includes people with MCI (unaware of their diagnoses), and people with dementia (with whom diagnoses were shared). We attempted to overcome methodological limitations present in past studies analysing cognitive impairment and health care use. We employed panel data on participants from the Sydney Memory and Ageing Study linked to their administrative data on health care use to enable objective and accurate costing. We accounted for a far more detailed set of covariates than has been used in past studies through mapping self-reported interview data to categories in Andersen's Expanded Behavioural Model of health services use (Services Australia, 2022). Two-part models were employed for estimation, and the panel element of the study was exploited by estimating ‘annual’ costs incurred in the year after the interviews and assessments.

We also delved further than past studies into potential moderators of health care use for those with MCI, conducting subgroup analyses by demographic characteristics and other characteristics of interest that may moderate health care use, including level of carer support. We undertook these subgroup analyses based on past US study findings showing potential service gaps for people with cognitive impairment in the community which past authors posit may relate to carer support, underdiagnosis and an inability to identify or communicate symptoms [ (Caspi et al., 2009), (Callahan et al., 1995), (Ton et al., 2017), (Walsh et al., 2003)]. No past explorative work on moderators has been conducted specifically for the older Australian community with cognitive impairment.

Our overall purpose in this study was to explore medical and pharmaceutical use patterns for older people with diagnosed and undiagnosed cognitive impairment, to generate primary care policy implications for future diagnosis and management of MCI in the Australian community.

## 2 METHODS

### 2.1 Data

#### 2.1.1 Study cohort

For our analyses, we used the respondent sample from the Sydney Memory and Ageing Study (MAS), a longitudinal study which commenced in 2005 to investigate clinical characteristics, prevalence, and changes in cognitive impairment in a community sample, with follow-ups occurring biennially. Community-dwelling individuals without dementia (N = 1037) aged 70–90 years were recruited randomly through the electoral roll from two adjacent federal areas in New South Wales (Kingsford-Smith and Wentworth), with Wave 1 data collection conducted between September 2005–November 2007 (Sachdev et al., 2010). The study was approved by the ethics committees of the University of New South Wales (UNSW) Australia and the South-Eastern Sydney and Illawarra Area Health Service. To be eligible, participants needed to be sufficiently fluent in English to complete comprehensive psychometric assessments.

**Fig. 1:**
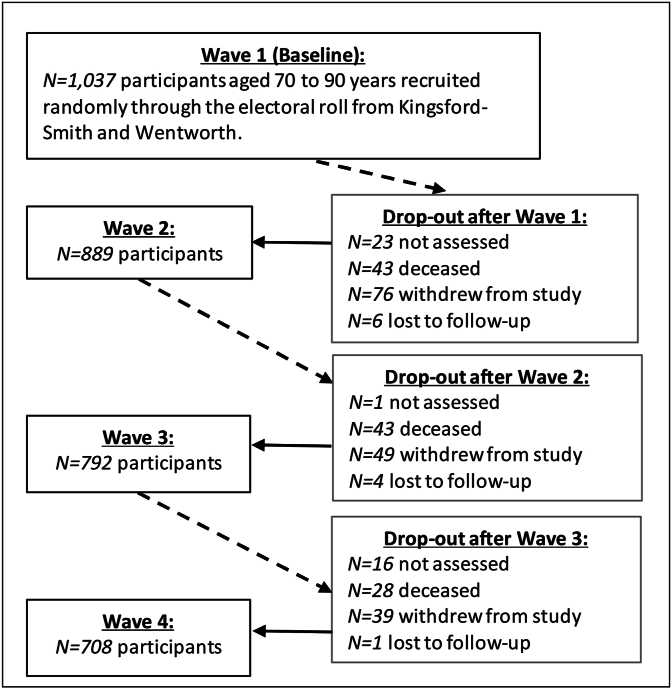
Attrition and sample sizes over four waves of the Sydney Memory and Ageing Study.

We analysed four waves of the MAS representing approximately six years of follow-up data after the baseline survey (Wave 1). Fig. 1 shows sample sizes and attrition over four waves of the MAS. By Wave 4, 329 individuals of the original sample of 1037 (≈32%) had dropped out of the study due to death, withdrawal and being lost to follow-up, with some individuals unable to undergo the needed study assessments. High attrition is common amongst studies in the elderly population, particularly for individuals in poorer health. In Section 2.2.2, we discuss our chosen approach for adjusting modelling estimates to account for this attrition in the MAS panel, and the underlying assumption of attrition being ‘missing at random’ after controlling for covariates.

#### 2.1.2 Outcome measures: medical and pharmaceutical costs

Consent was obtained from MAS participants at baseline (Wave 1) to link to their survey data to their administrative data on government-funded medical and pharmaceutical use over the MAS study period. All participants were subsequently linked to their Medicare records (2005–2014). Medicare is Australia's national health insurance scheme that provides Australians with free or subsidised health care. The Australian Government plays a direct financing role with respect to medical services in Australia, with Medicare financed from taxation revenue (Services Australia, 2022).

Medicare records include data on medical care and pharmaceutical payments made by patients and the government under the Medicare Benefits Schedule (MBS) and the Pharmaceutical Benefits Scheme (PBS). Most Medicare services (83 per cent) are bulk-billed, where the provider charges exactly the government-paid benefit and there is no patient co-payment. Appendix A presents detailed information on the MBS and PBS, subsidies available to older people and some caveats related to the estimation of PBS costs.

We estimated total MBS and PBS costs incurred by participants outside of hospitals (non-hospital medical costs), calculated as patient co-payments plus benefits payable by the Australian Government, in the one year after the interview dates for each wave. The cost perspective taken in this study is a health care system perspective, including annual expenditure incurred by the Australian Government and individuals. Other funders within the Australian health care system include private health insurers and state governments. However, within the context of medical services, these funders play a role if these costs are incurred within a hospital setting (public or private). Since we focus on out-of-hospital medical costs, capturing costs incurred by the Australian Government and individuals encompasses a health care system cost perspective.

Within our study, participants with no MBS or PBS services billed in the year after a particular survey wave were imputed as having ‘zero’ costs. In addition to looking at total annual MBS costs, we also analysed broad MBS cost categories related to the diagnosis and medical management of early dementia and cognitive impairment, including ‘Professional attendances’ (which includes attendances with general practitioners, specialist geriatricians, neurologists, psychiatrists), ‘Diagnostic imaging’ (which includes items such as magnetic resonance imaging or computed tomography), and ‘Pathology’ (Australian Institute of Health and Welfare, 2021a). MBS cost categories that we did not separately estimate (broadly unrelated to the diagnosis and medical management of early dementia and cognitive impairment) included ‘Therapeutic procedures’ (including surgical), ‘Oral and maxillofacial services’, ‘Diagnostic procedures’, ‘Dental services’ and ‘Miscellaneous services’ (including allied health).

Medications used to manage the severity and progression of cognitive and behavioural symptoms and improve quality of life in dementia include acetyl cholinesterase inhibitors and a *N*-methyl-d-aspartate (NMDA) receptor antagonist (Australian Institute of Health and Welfare, 2019). In addition to total annual PBS costs, we therefore also estimated pharmaceutical costs specifically falling under the broad PBS grouping of ‘nervous system drugs’ which contain these medications [ (Department of Health, 2022a, 2022b]. All estimated costs were inflated to Australian dollars in 2023 (AUD$), using annual health inflation rates from the Australian Institute of Health and Welfare (Australian Institute of Health and Welfare, 2021b) and the Consumer Price Index (health system) for more recent years (Australian Bureau of Statistics, 2023).

#### 2.1.3 Key covariates of interest: cognitive impairment z-score and diagnoses

Over MAS waves 1–4, a comprehensive neuropsychological test battery was administered to participants. These tests measured the five cognitive domains of memory, language, attention/processing speed, visuospatial, and executive functioning (Brodaty et al., 2013). A composite z-score for global cognition was computed by averaging the individual domain scores (see Appendix B Table B1 for more details). We used this global z-score as one of the key covariates of interest and a continuous measure in our modelling, with a unit change in the z-score representing ±1 standard deviation in cognition. We also included the z-score's squared term to account for non-linearity in the relationship between cognition score and costs.

The baseline method for constructing the z-score assumes equal weights across cognitive domains. As a robustness check, we used an alternative method for constructing the z-score by calculating the first (principal) factor from the battery of cognitive tests using principal components analysis and examining the effect on modelling results (Section 3.3).

The alternative covariates of interest were official diagnoses of dementia and MCI determined at each wave, which were explored through a second set of estimations. An expert panel diagnosed MCI in the MAS using international consensus criteria for participants with complete neuropsychological and functional performance data and adequate English proficiency (Sachdev et al., 2010). MCI diagnosis was based on the presence of objective cognitive impairment (less than −1.5 SD in one domain), subjective complaints about cognition and memory and the absence of significant functional impairment (Brodaty et al., 2017). Within the MAS, participants and their general practitioners were contacted via feedback letters if they were newly diagnosed with dementia by a consensus panel of three or more specialist clinicians.

MCI diagnoses made within community samples are quite different to those made within clinics, with substantially higher rates of reversion to normal cognition (Brodaty et al., 2013). For this reason, the MAS did not share MCI diagnoses with participants or informants. Hence, the estimated costs associated with MCI essentially applied to a study population unaware that they had MCI (‘undiagnosed’). Since the needed criteria for determining diagnoses was more expansive than just the objective testing (subjective complaints, expert panel agreement and objective tests), the sample sizes for the diagnoses estimations were also smaller than those in the z-score estimations.

#### 2.1.4 Other covariates

Our study mapped self-reported interview variables to categories in Andersen's Expanded Behavioural Model of health services use (Andersen, 1995). Covariate selection was informed through a review of past literature on factors associated with health care use in older adults and the cognitively impaired.

Since comorbidities are clearly associated with age-related cognitive impairment and dementia (Caracciolo et al., 2013; Fabbri et al., 2016; Vassilaki et al., 2015), we controlled for the total number of diagnosed comorbidities at each wave, using an extensive self-reported list captured in the MAS (e.g. diabetes, high blood pressure, kidney disease, heart problems). Other covariates controlled for included education level [ (Caspi et al., 2009), (Jenkins et al., 2016), (Zhu et al., 2013), (Luppa et al., 2008)], self-reported health status measured through the Assessment of Quality of Life 6 dimensions (AQOL-6D) to proxy perceived ‘need’ for health care [ (Mossey & Shapiro, 1982), (Reuben et al., 1992)], self-reported physical activity participation, and smoking and drinking behaviour. The AQOL-6D, in particular, conceptualises quality of life in terms of ‘handicap’ or the impact of self-reported health on personal functioning and satisfaction within a person's social context [ (Mossey & Shapiro, 1982), (Reuben et al., 1992)] and is therefore likely to be a good proxy of perceived health care need for older people.

While some past studies have used measures of socioeconomic status as control variables [ (Caspi et al., 2009), (Aigbogun et al., 2017), (Ton et al., 2017), (Walsh et al., 2003)], MAS did not collect data on participant income and net worth. Nonetheless, the sample was recruited from two adjacent federal government areas in New South Wales, with a similar, high socioeconomic status (Parliament of Australia, 2010). Additionally, we controlled for past years of education and occupation type to proxy for any residual differences between participants in income and wealth that might influence health care use.

The final list of covariates (Table 1) broadly covered ‘predisposing’, ‘enabling’, ‘need’ and ‘personal health practices’ categories in Andersen's Expanded Behavioural Model of health services use [ (Andersen, 1995), (Travers et al., 2020), (Evashwick et al., 1984)] (see Appendix B Table B3). Since our estimations were based on a panel, some participants dropped out of the sample due to death and attrition. As death may confound the estimation of health care costs, we included a dummy variable to capture individuals who dropped out due to death during the study (Mora et al., 2015). Data about participants' date and cause of death were obtained from the New South Wales Registry of Births, Deaths, and Marriages.

**Table 1:** Covariate and z-score averages by diagnosis category (pooled observations - waves 1–4).

	Sample sizes and means						Two sample *t*-test for difference in means		
	Normal cognition		MCI		Dementia		*Normal vs MCI*	Normal vs dement.	*MCI vs dement.*
	N	mean	N	Mean	N	mean	Test-stat.	Test-stat.	Test-stat.
*Z-score*	1948	0.193	953	−0.890	104	−2.697	29.052	19.233	11.856
*Covariates:*									
Age (years)	1817	80.03	984	80.88	158	84.96	−4.237	−12.298	−9.752
Female	1817	0.585	984	0.503	159	0.503	4.118	1.959	−0.002
Death during study	1817	0.061	984	0.103	159	0.075	−3.698	−0.649	1.183
Education (years)	1817	11.803	984	11.583	158	12.065	1.615	−0.804	−1.470
Married/de-facto	1817	0.402	984	0.390	159	0.377	0.669	0.637	0.304
Home duties/other	1817	0.053	984	0.069	159	0.063	−1.614	−0.463	0.303
Managers/Professionals	1817	0.534	984	0.485	159	0.516	2.436	0.441	−0.700
Clerical and services	1817	0.349	984	0.331	159	0.252	1.037	2.694	2.082
Transport/trades/labour	1817	0.063	984	0.115	159	0.170	−4.456	−3.512	−1.734
English-speaking	1817	0.903	984	0.965	159	0.786	−6.843	3.511	5.408
Comorbidities (number)	1817	11.099	984	11.384	159	13.912	−1.003	−5.237	−4.535
Alcohol consumption (drinks/wk)	1801	2.872	975	2.899	135	2.207	−0.273	4.071	4.074
Smoke every day	1810	0.282	961	0.303	110	0.300	−1.180	−0.401	0.074
Participates in physical activity	1809	0.671	982	0.663	159	0.635	0.406	0.886	0.673
AQOL-6D	1724	0.824	911	0.804	99	0.702	3.332	6.030	4.956

### 2.2 Modelling approach

We modelled the costs associated with the continuous global z-score, MCI and dementia diagnoses using a two-part model (2PM) panel data approach, separating modelling the probability of health care use from the intensity of health care use (Walsh et al., 2003). The 2PM is widely employed in health economics and health services research for estimating health care costs (Mullahy, 1998), including those associated with cognitive impairment (Walsh et al., 2003). It simultaneously accounts for masses of zero observations (i.e. those incurring no health care costs), strongly skewed distributions and long right-hand tails (i.e. individuals making heavy use of health care) (Deb & Norton, 2018). While the first part of a 2PM is estimated for the entire sample (whether individuals incur health care costs or not), the second part of the 2PM is estimated only for the sample of individuals who incur some costs (i.e. ‘non-zero’ observations).

In our data, 11.7 per cent of participants incurred zero MBS costs over waves 1–4, 13.2 per cent incurred zero PBS costs and higher proportions incurred zero costs for specific MBS/PBS cost items (32–46 per cent). The cost distributions were also heavily skewed with long right-hand tails, making these suited to 2PM estimation (see Appendix Figure B1).

We modelled the first part (probability of incurring any costs in the year following each wave) using a random-effects logit model. Here, (1) represents the equation using z-score as a covariate of interest, and (2) represents the equation using diagnoses as the covariate of interest:

(1) $$\text{logit}\left(P_{it}\right)=\beta_{0}+\beta_{1}z_{it}+\beta_{2}z_{it}^{2}+\emptyset X_{it}{+\alpha }_{i}$$

(2) $$\text{logit}\left(P_{it}\right)=\beta_{0}+\gamma_{1}\;{MCI}_{it}+\gamma_{2}\;{Dementia}_{it}+\emptyset X_{it\;}+\alpha_{i}$$

where $P_{it}$ is the probability of individual i incurring health care costs $Y_{it}$ after survey wave t;

logit ($P_{it})$ is the log-odds of individual i incurring health care costs after survey wave t; $\beta_{0}$ is a constant term; $\beta_{1}$ and $\beta_{2}$ are the estimated coefficients for the effects of a change in z-score and the squared term for z-score, respectively, on the log-odds of incurring costs; $\gamma_{1}$ and $\gamma_{2}$ are the estimated coefficients for the effects of an MCI diagnosis and dementia diagnosis, respectively, on the log-odds of incurring costs (relative to normal cognition); ∅ is a vector of estimated coefficients representing the effects of all the other covariates (Section 2.1.4) on the log-odds of incurring costs; and $\alpha_{i}$ is the individual-specific random effect, capturing unobserved, individual-level heterogeneity.

The second part of the model (dollar amount incurred in the year following the survey wave) was estimated using a generalised estimating equations (GEE) regression (Mora et al., 2015) for observations with non-negative costs. A Box-Cox (Deb et al., 2017) and modified Park test (Park, 1966) determined a natural log transformation and gamma distribution to be the appropriate link function and distribution family, respectively, for the GEE.

A GEE approach was chosen over other panel data modelling approaches since it accommodates non-normal (skewed) distributions and accounts for correlated, repeated measures over time. We chose random effects, exchangeable within-panel correlation structure to account for within-individual correlation across the four MAS waves.

An alternative modelling approach would have been to use individual-level fixed effects estimation to control for potential heterogeneity bias from time constant, individual-level unobservables such as personality traits. However, this approach would also remove the individual-specific, time-invariant contribution of genes and hereditary factors related to cognitive impairment. This was not deemed to be appropriate to the purposes of our study.

Equations for the second part of the model are shown below, with (3) representing the equation using z-score as a covariate of interest, and (4) representing the equation using diagnoses as the covariate of interest:

(3) $$g\left(\mu_{it}\right)=\beta_{0}+\beta_{1}z_{it}+\beta_{2}z_{it}^{2}+\emptyset X_{it}+\alpha_{i}$$

(4) $$g\left(\mu_{it}\right)=\beta_{0}+\gamma_{1}\;{MCI}_{it}+\gamma_{2}\;{Dementia}_{it}+\emptyset X_{it}+\alpha_{i}$$

where g (.) is the natural log transformation; and.

$\mu_{it}$ is the mean of the gamma-distributed health care costs $Y_{it}$, with other terms the same as those presented in equations (1), (2).

The variance function for the gamma-distributed health care costs $Y_{it}$ is:

$Var\left(Y_{it}\right)=\frac{\mu_{it}^{2}}{\alpha }$ where α is the shape parameter of the gamma distribution.

In the results section, we present average marginal effects (partial derivatives) for both parts of the models, rather than the modelling coefficients, to enhance interpretability. The marginal effects convey the average effects on the probability and intensity of health care use from changes in the cognitive z-score and diagnoses of MCI or dementia during MAS. With continuous z-score as the covariate of interest, for instance, the marginal effect of a change in the z-score on health care use varies along the z-score distribution (Appendix Figure B4). Within our results, therefore, we present the ‘average’ marginal effect from each observation across the entire distribution. For transparency, the modelling coefficients for the covariates of interest are also included in Appendix Tables B2 and B3.

All estimations were performed using Stata 16 (StataCorp LLC, College Station, TX). The sample size varied between 2676–3010 observations (955–1009 individuals) for the first-part random effects logit estimation, and 1153–2596 observations (551–887 individuals) for the second-part GEE estimation.

#### 2.2.1 Differences in predicted costs

Total predicted costs for those with MCI, dementia and normal cognition were generated by multiplying predicted probabilities from the first part of the model by predicted costs from the second part of the model. Predictions were generated for each individual in the sample, and then averaged over the entire sample (Au, 2012). Standard errors for predictions were calculated using the bootstrap method (1000 replications). Average predicted cost differences for dementia versus normal cognition, and MCI versus normal cognition were than calculated, and are presented in Section 3.4.

#### 2.2.2 Attrition-adjusted estimation

As noted in Section 2.1.1, approximately 32% of individuals present at baseline had dropped out of the MAS by Wave 4 due to death, drop-out and withdrawal from the study. This creates successive ‘missing data’ over time, with some individuals in the estimations having data across all waves while others contribute data for some (earlier) waves. In surveys of older people, many drop-outs are likely to be related directly to health (i.e. serious illness or death), with those remaining in the panel over time likely to be healthier on average (Jones et al., 2006).

While we used the entire unbalanced panel for the baseline estimations, we examined the potential effects of attrition over time on the estimates, by also looking at the estimates using only ‘complete cases’ in a balanced panel (i.e. those who were present over all four MAS waves).

To adjust the modelling estimates for potential bias from non-response, we adopted an inverse probability weighted (IPW) estimator and applied it to both parts of the model (Jones et al., 2006). This approach assumes the notion of ‘missingness-at-random’, or ignorable non-response after adjusting for covariates, including health-related variables, associated with non-response. To apply an IPW approach, we estimated logistic regression models for non-response (i.e. ‘drop-out’) at each wave, conditional on individual characteristics measured at the first wave.^1^ These estimations showed education level and health-related variables^2^ to be significantly associated with dropping out of the survey over time [ (Lipnicki et al., 2017), (Lipnicki et al., 2013)].

The inverse fitted response probabilities were used to weight all observations in the two-part models.^3^ The IPW estimator aims to recreate a representative sample of the initial cohort by weighting more heavily those individuals with a high probability of non-response, who are ‘underrepresented’ in the observed sample. In the results section (Section 3), we present the original estimates alongside the balanced sample and attrition adjusted IPW estimates, to enable comparisons.

#### 2.2.3 Subgroup analyses

To examine potential moderators of health care use for people with MCI, we conducted subgroup analyses for the first and second parts of the model using interaction terms between certain characteristics of interest and a dummy variable indicating MCI diagnosis. Within these analyses, we explored differences in the probability and intensity of incurring costs by sociodemographic characteristics (gender, occupation) and other characteristics of interest including level of carer support for various activities. For the subgroup analyses, both original and attrition adjusted modelling coefficients are presented (Section 3.5).

## 3 RESULTS

### 3.1 Descriptive statistics for covariates

Our two covariates of interest (z-score and diagnoses) broadly aligned with each other (see Appendix B Figures B2-B3). Patients diagnosed with dementia were more likely to have z-scores below −2 (Appendix B Figure B3). As expected, there were statistically significant differences in z-score between those with normal cognition, dementia, and MCI, indicated by the two-sample *t*-test statistics presented in Table 1.

Overall, people with normal cognition, MCI and dementia in the sample differed significantly across many covariates, excluding marital status, years of education, smoking behaviour, and physical activity participation (|*t|< 1.96)*. Those with dementia had a significantly higher average number of comorbidities than those with MCI and normal cognition, and lower AQOL-6D scores.

### 3.2 Estimation results

The estimated marginal effects for the first and second parts of the model are presented for the z-score and diagnoses estimations in Table 2 and Table 3, respectively. For the z-score estimations (panels A and C), the marginal effects represent the per cent change in the probability of incurring costs in the year after a survey wave (first part), and the dollar change in costs for those who incurred costs (second part), associated with a 1 standard deviation change in z-score. For the diagnoses estimations (panels B and D), the marginal effects represent the percent change in the probability of incurring costs in the year after a survey wave (first part), and dollar change in costs for those who incurred costs (second part), for people diagnosed with either MCI or dementia, relative to people with normal cognition.

**Table 2:** Part one model results – Marginal effects - percentage point change in probability of health care use associated with z-score and diagnoses.

(A) Z-score									
	*(A1) Unbalanced sample (random effects logit)*			*(A2) Balanced sample (random effects logit)*			*(A3) IPW-weighted (pooled logit)*		
	*Effect*	*S.E*	*P*	*Effect*	*S.E*	*P*	*Effect*	*S.E*	*P*
1 S.D ↑ in z-score:									
All MBS	0.020***	0.006	0.001	0.015**	0.007	0.031	0.017**	0.007	0.010
*N (individuals)*	*3010 (1009 individuals)*			*2512 (703 individuals)*			*2911 (965 individuals)*		
Prof. Attend. (MBS)	0.028***	0.009	0.002	0.021**	0.010	0.029	0.026***	0.009	0.003
*N (individuals)*	*3010 (1009 individuals)*			*2512 (703 individuals)*			*2911 (965 individuals)*		
Diag. Imaging (MBS)	0.022**	0.010	0.024	0.014	0.011	0.199	0.023**	0.010	0.027
*N (individuals)*	*3010 (1009 individuals)*			*2512 (703 individuals)*			2911 (965 individuals)		
Pathology (MBS)	0.025***	0.009	0.009	0.023**	0.010	0.027	0.023**	0.009	0.010
*N (individuals)*	*3010 (1009 individuals)*			*2512 (703 individuals)*			*2911 (965 individuals)*		
All PBS	0.018**	0.008	0.022	0.013	0.008	0.121	0.012*	0.007	0.090
*N (individuals)*	*3010 (1009 individuals)*			*2512 (703 individuals)*			2911 (965 individuals)		
Nerv. Syst. Drugs (PBS)	−0.027**	0.011	0.019	−0.028**	0.013	0.030	−0.027***	0.010	0.005
*N (individuals)*	*3010 (1009 individuals)*			*2512 (703 individuals)*			2911 (965 individuals)		
(B) Diagnoses [Reference category: Normal cognition]									
	*(B1) Unbalanced sample (random effects logit)*			*(B2) Balanced sample (random effects logit)*			(B3) IPW-weighted (pooled logit)		
	*Effect*	*S.E*	P	Effect	S.E	P			
All MBS									
MCI	−0.019*	0.011	0.078	−0.017	0.012	0.172	−0.025*	0.014	0.087
Dementia	0.011	0.025	0.664	0.019	0.023	0.407	0.042	0.031	0.172
*N (individuals)*	*2676 (955 individuals)*			*2262 (686 individuals)*			2585 (915 individuals)		
Prof. Attend (MBS)									
MCI	−0.026	0.018	0.156	−0.021	0.019	0.253	−0.026	0.018	0.153
Dementia	0.155***	0.055	0.005	0.116**	0.054	0.031	0.175***	0.052	0.001
*N (individuals)*	*2676 (955 individuals)*			*2262 (686 individuals)*			2585 (915 individuals)		
Diag. Imag. (MBS)									
MCI	−0.037*	0.019	0.060	−0.029	0.021	0.166	−0.033*	0.019	0.090
Dementia	0.063	0.056	0.262	0.094	0.059	0.113	0.087	0.058	0.133
*N (individuals)*	*2676 (955 individuals)*			*2262 (686 individuals)*			2585 (915 individuals)		
Pathology (MBS)									
MCI	−0.032*	0.018	0.077	−0.028	0.020	0.154	−0.032*	0.019	0.084
Dementia	0.210***	0.056	0.000	0.160***	0.055	0.003	0.217***	0.056	0.000
*N (individuals)*	*2676 (955 individuals)*			*2262 (686 individuals)*			2585 (915 individuals)		
All PBS									
MCI	−0.010	0.013	0.427	−0.012	0.014	0.381	−0.024	0.015	0.105
Dementia	0.002	0.038	0.965	−0.000	0.037	1.000	0.016	0.042	0.708
*N (individuals)*	*2676 (955 individuals)*			*2262 (686 individuals)*			2585 (915 individuals)		
Nerv. Syst. (PBS)									
MCI	−0.014	0.020	0.478	−0.015	0.022	0.498	0.012	0.020	0.551
Dementia	0.131**	0.057	0.022	0.140	0.062	0.025	0.206***	0.061	0.001
*N (individuals)*	*2676 (955 individuals)*			*2262 (686 individuals)*			2585 (915 individuals)		

* Full modelling results for all covariates available from the authors. P < 0.1*, p < 0.05**, p < 0.01***.

**Table 3:** Part two model results – Marginal effects - $ change in costs incurred for those who incurred any costs associated with z-score and diagnoses.

(C) Z-score									
	(C1) Unbalanced sample GEE			(C2) Balanced sample GEE			(C3) IPW-weighted GEE		
	Effect	S.E	P	Effect	S.E	P	Effect	S.E	P
1 S.D ↑ in z-score:									
All MBS	−130.16	100.41	0.195	−140.49	113.45	0.216	−94.29	114.85	0.412
N (individuals)	2596 (885 individuals)			2245 (655 individuals)			2508 (844 individuals)		
Prof. Attend. (MBS)	−101.49***	35.26	0.004	−110.75***	37.45	0.003	−95.26**	39.98	0.011
N (individuals)	1794 (734 individuals)			1661 (624 individuals)			1707 (693 individuals)		
Diag. Imaging (MBS)	52.47	36.94	0.156	44.74	38.99	0.251	67.75	52.95	0.201
N (individuals)	1481 (688 individuals)			1372 (595 individuals)			1409 (652 individuals)		
Pathology (MBS)	−30.47*	15.94	0.056	−32.51*	17.09	0.057	−26.75*	16.s19	0.098
N (individuals)	1610 (709 individuals)			1487 (606 individuals)			1532 (672 individuals)		
All PBS	−155.78*	94.29	0.099	−185.57*	98.20	0.059	−147.95	167.20	0.376
N (individuals)	2547 (887 individuals)			2191 (654 individuals)			2460 (847 individuals)		
Nerv. Syst. Drugs	−92.29***	8.77	0.000	−71.97***	11.51	0.00	−57.07***	9.28	0.000
N (individuals)	1291 (589 individuals)			1093 (445 individuals)			1238 (562 individuals)		
(D) Diagnoses [Reference category: Normal cognition]									
	(D1) Unbalanced sample GEE			(D2) Balanced sample GEE			(D3) IPW-weighted GEE		
	Effect	S.E	P	Effect	S.E	P			
All MBS									
MCI	72.28	208.05	0.728	121.31	233.06	0.603	92.45	254.19	0.716
Dementia	−107.93	459.87	0.814	32.92	518.25	0.949	−75.72	610.24	0.901
N (individuals)	2307 (835 individuals)			2011 (634 individuals)			2226 (799 individuals)		
Prof. Attend (MBS)									
MCI	4.94	73.04	0.946	29.91	76.58	0.696	11.72	77.19	0.879
Dementia	338.94**	153.03	0.027	426.29**	164.79	0.010	388.26	299.66	0.195
N (individuals)	1609 (697 individuals)			1502 (606 individuals)			1531 (662 individuals)		
Diag. Imag. MBS)									
MCI	−120.22	75.17	0.110	−101.04	79.32	0.203	−130.97	80.19	0.102
Dementia	−192.29	158.36	0.225	−155.15	169.06	0.359	−198.96	155.75	0.201
N (individuals)	1324 (645 individuals)			1236 (568 individuals)			1259 (613 individuals)		
Pathology (MBS)									
MCI	6.85	34.26	0.842	8.13	36.23	0.823	1.03	40.69	0.980
Dementia	−33.83	69.12	0.625	−4.75	75.42	0.950	−24.03	98.74	0.808
N (individuals)	1450 (674 individuals)			1350 (589 individuals)			1380 (642 individuals)		
All PBS									
MCI	−47.49	150.51	0.752	127.06	161.16	0.430	−40.95	176.56	0.817
Dementia	1899.51***	197.36	0.000	1378.23***	248.22	0.000	1895.6***	629.8	0.003
N (individuals)	2265 (835 individuals)			1962 (630 individuals)			2185 (799 individuals)		
Nerv. Syst. (PBS)									
MCI	68.09**	29.46	0.021	68.41***	24.99	0.006	61.58	40.33	0.127
Dementia	361.68***	28.24	0.000	331.68***	25.02	0.000	361.14***	46.26	0.000
N (individuals)	1153 (551 individuals)			979 (422 individuals)			1153 (551 individuals)		

* Full modelling results for all covariates available from the authors. P < 0.1*, p < 0.05**, p < 0.01***.

The unbalanced sample, balanced sample and IPW (attrition-adjusted) results for the probability of incurring costs are presented in Tables 2 and 3. Generally, the statistical significance of the estimated marginal effects aligns across the unbalanced sample, balanced sample and IPW estimates, with some effects becoming insignificant for the balanced sample estimation. The differences in magnitude between the unbalanced and balanced sample estimates suggests that accounting for attrition in modelling is appropriate.

While there are slight differences in effect magnitude, there is consistency in the significance and direction of the estimated effects across the unbalanced sample and attrition adjusted estimates. We therefore present the unbalanced sample and attrition-adjusted estimates as the ‘bounds’ for the marginal effects in our results discussion, and only discuss results that are statistically significant in the baseline and after attrition-adjustment.

#### 3.2.1 Annual probability of incurring costs (use)

Overall, a one standard deviation increase in cognition was significantly associated with an increased probability of incurring any MBS and PBS costs in the year following a survey wave (Panel A1-A3 in Table 2). Improved cognition was also associated with an increased probability of incurring costs across all the specific MBS categories examined, including professional attendances (2.6–2.8 percentage points), diagnostic imaging (2.2–2.3 percentage points), and pathology (2.3–2.5 percentage points). Conversely, for the specific PBS category examined, nervous system drugs, improved cognition was associated with a decreased probability of incurring this cost type (−2.7 percentage points).

Nonetheless, these ‘average marginal effect’ results mask that the direction and significance of the effects varies across the z-score cognitive spectrum. To explore this further, Appendix Figure B4 plots the marginal effects and confidence intervals for each cost type at specific values of the z-score and shows that at the more severe end of the spectrum (z < −2), improved cognition was associated with significantly *reduced* probabilities of incurring professional attendances, diagnostic imaging, and pathology costs (negative marginal effects). Wide confidence intervals at the extreme ends of the spectrum (due to small sample sizes), reduce the ability to detect significance.

The z-score results broadly align with the diagnoses results in Panels B and D. People diagnosed with MCI during MAS had a small but significantly reduced probability (at a 10% level of significance) of incurring any MBS costs (−1.9-2.5 percentage points) relative to people with normal cognition in the year following a survey wave. People diagnosed with MCI also had significantly reduced probabilities of incurring costs across the specific MBS categories analysed including diagnostic imaging (−3.3-3.7 percentage points) and pathology (−3.2 percentage points) relative to normal cognition.

Other cost categories (total PBS costs, professional attendances, and nervous system drugs) were not significantly different between MCI and normal cognition. People diagnosed with dementia during MAS had a significantly increased probability of incurring professional attendances (15.5–17.5 percentage points), pathology (21.0–21.7 percentage points), and nervous system drugs costs (13.1–20.6 percentage points) in the year following a survey wave, relative to cognitively normal individuals.

#### 3.2.2 Annual amount of costs incurred

Panels C1–C3 in Table 3 shows that, for the participant sample that incurred costs in the year after a survey wave, a one standard deviation increase in cognition was associated with reduced amounts of annual professional attendances (-AUD$101–95), pathology (-AUD$30–27) and nervous system drugs (-AUD$92–57) costs.

A dementia diagnosis (Panel D1-D3) was associated with an increase in annual PBS (AUD$1896–1900) and nervous system drugs costs (AUD$361–362), relative to normal cognition. While an MCI diagnosis was associated with significantly increased annual nervous system drugs costs relative to normal cognition for the unbalanced sample estimation (D1), this result became insignificant after attrition adjustment.

### 3.3 Robustness check: constructing z-score using principal components analysis

The baseline method for constructing the continuous z-score assumed equal weights across all cognitive test domains. As a robustness check, we used an alternative method for constructing the z-score with the first (principal) factor from the cognitive test battery using principal components analysis, since performance across cognitive tests is likely to be significantly correlated (Lyall et al., 2016).

The cognitive z-scores constructed using the two methods were highly positively correlated across the MAS respondent sample (correlation coefficient = 0.95). The estimation results (average marginal effects) for the first and second modelling parts using the principal components analysis z-scores are presented in Appendix Tables B4 and B5 (original and attrition-adjusted estimates). The statistical significance and direction of these estimated marginal effects align strongly with the baseline results, although the effect magnitude (effect size) is noticeably larger in the results using the principal components analysis z-score. Overall, we present the more conservative baseline results as the main results of this study and conclude that our study findings (based on statistical significance) are robust to the method used to construct the z-scores.

**Table 4:** Subgroup analysis through interactions with MCI – Part 1 coefficients [Reference: normal cognition].

	*(B1) Unbalanced sample (random effects logit)*			*(B3) IPW-weighted (pooled logit)*		
	*Coeff.*	*S.E*	*P*	*Coeff.*	*S.E*	*P*
*All MBS*						
MCI Male	−0.719	0.495	0.146	−0.196	0.181	0.280
MCI Female	−0.430	0.378	0.256	−0.251	0.179	0.161
MCI, Past occupation: Managerial/Professional)	−0.205	0.431	0.634	−0.108	0.180	0.546
MCI, Past occupation: Non-Managerial/Professional)	−0.829*	0.423	0.050	−0.351*	0.181	0.053
MCI, amnestic	−0.539	0.356	0.129	−0.205	0.154	0.184
MCI, non-amnestic	−0.602	0.423	0.155	−0.260	0.176	0.140
MCI, lives alone	−0.690*	0.412	0.094	−0.434**	0.174	0.012
MCI, does not live alone	−0.398	0.469	0.396	0.015	0.190	0.937
MCI, carer helps with making medical decisions	−0.411	0.557	0.461	−0.092	0.249	0.712
MCI, carer does not help with making medical decisions	−0.611*	0.346	0.077	−0.273*	0.149	0.067
MCI, carer helps with transport	−0.109	0.504	0.829	0.171	0.228	0.455
MCI, carer does not help with transport	−0.695**	0.347	0.045	−0.409***	0.155	0.008
*All PBS*						
MCI Male	−0.227	0.334	0.497	−0.167	0.176	0.343
MCI Female	−0.164	0.343	0.633	−0.229	0.168	0.175
MCI, Past occupation: Managerial/Professional)	−0.031	0.323	0.924	−0.311	0.161	0.053
MCI, Past occupation: Non-Managerial/Professional)	−0.365	0.367	0.319	−0.059	0.184	0.748
MCI, amnestic	−0.183	0.290	0.528	−0.212	0.144	0.141
MCI, non-amnestic	−0.225	0.336	0.504	−0.191	0.174	0.273
MCI, lives alone	−0.108	0.334	0.746	−0.346**	0.163	0.034
MCI, does not live alone	−0.287	0.354	0.418	−0.037	0.181	0.838
MCI, carer helps with making medical decisions	0.317	0.470	0.501	0.108	0.245	0.660
MCI, carer does not help with making medical decisions	−0.353	0.272	0.194	−0.301**	0.140	0.031
MCI, carer helps with transport	0.638	0.442	0.148	0.297	0.225	0.186
MCI, carer does not help with transport	−0.524*	0.277	0.059	−0.410***	0.145	0.005
*Professional attendances*						
MCI Male	−0.052	0.227	0.820	−0.080	0.142	0.574
MCI Female	−0.369*	0.197	0.061	−0.194	0.135	0.149
MCI, Past occupation: Managerial/Professional)	−0.251	0.214	0.241	−0.048	0.137	0.726
MCI, Past occupation: Non-Managerial/Professional)	−0.180	0.205	0.381	−0.235*	0.138	0.088
MCI, amnestic	−0.047	0.188	0.802	−0.028	0.124	0.823
MCI, non-amnestic	−0.436**	0.198	0.027	−0.288**	0.130	0.027
MCI, lives alone	−0.356*	0.211	0.091	−0.019	0.135	0.887
MCI, does not live alone	−0.089	0.208	0.668	−0.280**	0.141	0.047
MCI, carer helps with making medical decisions	0.151	0.271	0.579	0.022	0.181	0.905
MCI, carer does not help with making medical decisions	−0.347**	0.176	0.049	−0.199*	0.116	0.087
MCI, carer helps with transport	0.165	0.247	0.505	0.188	0.165	0.255
MCI, carer does not help with transport	−0.406**	0.178	0.023	−0.310**	0.121	0.010
*Diagnostic imaging*						
MCI Male	0.055	0.174	0.752	0.060	0.135	0.657
MCI Female	−0.490***	0.168	0.004	−0.360***	0.135	0.007
MCI (Past occupation: Managerial/Professional)	−0.248	0.176	0.159	−0.209	0.135	0.120
MCI (Past occupation: Non-Managerial/Professional)	−0.209	0.161	0.193	−0.114	0.133	0.390
MCI amnestic	−0.125	0.151	0.408	−0.065	0.120	0.589
MCI non-amnestic	−0.369**	0.164	0.025	−0.291**	0.128	0.023
MCI and lives alone	−0.398**	0.171	0.020	−0.283**	0.142	0.046
MCI and does not live alone	−0.079	0.164	0.629	−0.057	0.128	0.655
MCI and carer helps with making medical decisions	0.059	0.212	0.782	0.053	0.177	0.764
MCI and carer does not help with making medical decisions	−0.329**	0.144	0.022	−0.240**	0.113	0.033
MCI and carer helps with transport	0.150	0.195	0.442	0.192	0.161	0.232
MCI and carer does not help with transport	−0.410***	0.145	0.005	−0.339***	0.118	0.004
*Pathology*						
MCI Male	−0.113	0.191	0.554	−0.105	0.138	0.450
MCI Female	−0.333*	0.173	0.054	−0.221*	0.133	0.097
MCI (Past occupation: Managerial/Professional)	−0.278	0.180	0.122	−0.113	0.134	0.401
MCI (Past occupation: Non-Managerial/Professional)	−0.177	0.180	0.326	−0.220	0.135	0.104
MCI amnestic	−0.181	0.163	0.265	−0.122	0.121	0.313
MCI non-amnestic	−0.297*	0.172	0.084	−0.230*	0.128	0.072
MCI and lives alone	−0.224	0.184	0.223	−0.170	0.138	0.218
MCI and does not live alone	−0.233	0.178	0.191	−0.165	0.132	0.211
MCI and carer helps with making medical decisions	−0.146	0.241	0.545	−0.165	0.182	0.365
MCI and carer does not help with making medical decisions	−0.249	0.151	0.099	−0.160	0.113	0.155
MCI and carer helps with transport	−0.107	0.220	0.626	−0.026	0.164	0.873
MCI and carer does not help with transport	−0.286*	0.153	0.062	−0.236**	0.118	0.045
*Nervous system drugs*						
MCI Male	−0.201	0.239	0.400	−0.040	0.133	0.762
MCI Female	−0.057	0.245	0.817	0.129	0.120	0.282
MCI (Past occupation: Managerial/Professional)	−0.232	0.231	0.315	−0.057	0.128	0.657
MCI (Past occupation: Non-Managerial/Professional)	−0.022	0.252	0.931	0.158	0.124	0.204
MCI amnestic	−0.105	0.208	0.614	0.057	0.111	0.605
MCI non-amnestic	−0.168	0.221	0.448	0.036	0.120	0.762
MCI and lives alone	0.065	0.256	0.798	0.051	0.127	0.688
MCI and does not live alone	−0.300	0.227	0.186	0.042	0.124	0.736
MCI and carer helps with making medical decisions	−0.187	0.290	0.521	0.018	0.167	0.913
MCI and carer does not help with making medical decisions	−0.109	0.204	0.594	0.062	0.105	0.557
MCI and carer helps with transport	−0.052	0.263	0.842	0.245	0.155	0.114
MCI and carer does not help with transport	−0.170	0.209	0.417	−0.047	0.109	0.670

* Full modelling results for all covariates available from the authors. P < 0.1*, p < 0.05**, p < 0.01***.

### 3.4 Difference in average annual predicted costs between cognitively normal people and those diagnosed with MCI and dementia

**Fig. 2:**
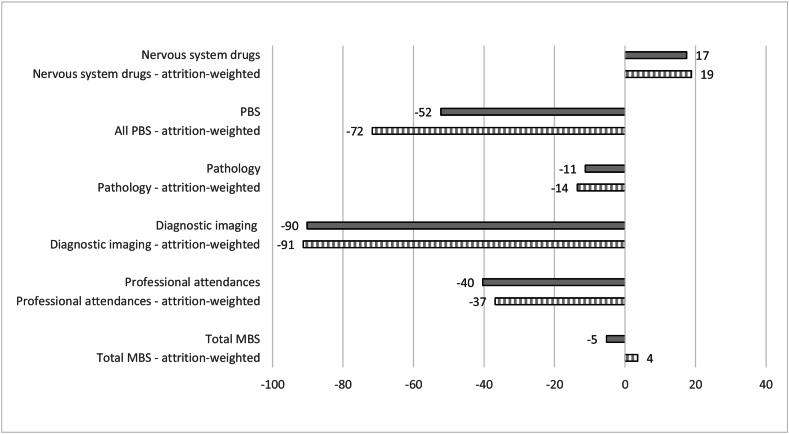
Differences in predicted annual costs ($AUD) for MCI versus normal cognition (pooled over four MAS waves).

**Fig. 3:**
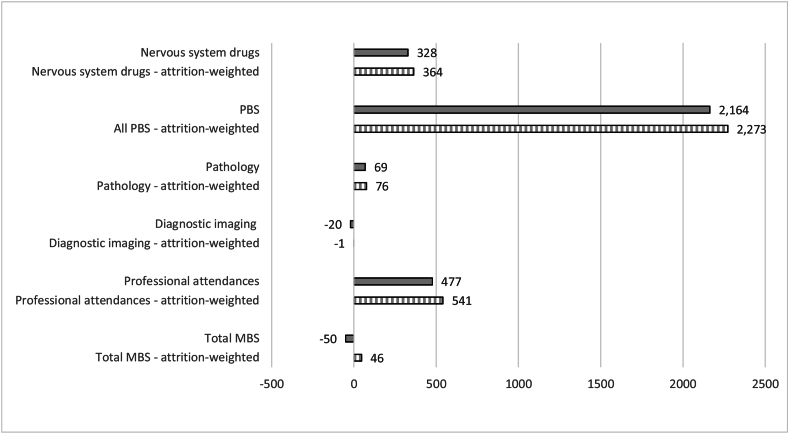
Differences in predicted annual costs ($AUD) for dementia versus normal cognition pooled over four MAS waves).

Fig. 2, Fig. 3 show significant differences in average annual predicted costs between people diagnosed with MCI and people with normal cognition, and people diagnosed with dementia and people with normal cognition. On average, people with MCI generally had significantly lower annual predicted costs across most cost categories than cognitively normal individuals, ranging from AUD$11–14 less in pathology costs to AUD$90–91 less in annual diagnostic imaging costs. However, people with MCI were predicted to have slightly *higher* average nervous system drugs (AUD$17–19) than cognitively normal individuals.

Differences were more substantial between people with diagnosed dementia and normal cognition. Those diagnosed with dementia during MAS were predicted to incur AUD$477–541 more in annual professional attendances costs, AUD$2164–2273 more in annual PBS costs and AUD$328–364 more in annual nervous system drugs costs than cognitively normal individuals. Total predicted annual MBS costs were not substantially different between people diagnosed with MCI and dementia, and those with normal cognition (with the predictions from unbalanced sample and attrition adjusted models having opposite signs). This suggests that other broad MBS categories not examined in this study (e.g. therapeutic procedures including surgical, miscellaneous etc.) likely offset the predicted cost differences across the MBS categories related to cognitive impairment.

### 3.5 Subgroup analysis: health care use for those with mild cognitive impairment

Past US studies have found potential outpatient service gaps for undiagnosed people with cognitive impairment living in the community [4 6 17 21], and suggested that these gaps may relate to underdiagnosis, an inability to identify or communicate symptoms, caregivers’ inability to identify needs or provide transport, or stigma.

Since no Australian studies have been conducted on outpatient service gaps for people with MCI, we conducted subgroup analyses for those with MCI exhibiting selected characteristics of interest including gender, past occupation (managerial/professional or not), amnestic versus non-amnestic MCI diagnosed during MAS, and living arrangements (live alone or not). We also conducted subgroup analyses by several variables related to the level of carer support available to individuals with MCI. Approx. 94% of MAS baseline participants had a carer or informant with 22% being spouses, 28% being children and 7% other relatives. Carers or informants self-reported whether they helped the participant with various tasks, including transportation and making medical decisions (subgroup sample sizes are presented in Appendix Table B6).

The subgroup characteristics can be seen as either ‘predisposing’ (e.g. gender), ‘need’ (amnestic versus non-amnestic symptoms) or ‘enabling’ factors (e.g. carer support, living arrangements, past occupation) under Andersen's Expanded Behavioural Model of health services use (Services Australia, 2022), and may therefore act as potential moderators of the relationship between MCI and health care use.

The modelling coefficients by MCI subgroup for the first and second parts of the models (unbalanced sample and attrition-adjusted) are presented in Table 4, Table 5. Most statistically significant subgroup results, consistent across the baseline and attrition-adjusted estimates, fell within the first part of the model related to probability of health care use. Overall, significantly reduced annual probabilities of incurring MBS costs (relative to normal cognition) were estimated for participants with MCI who lived alone, whose carer did not help with making medical decisions or transportation, and those with non-amnestic cognitive symptoms, relative to no significant reductions for those with MCI without these characteristics. This pattern was found across MBS categories including professional attendances, diagnostic imaging, and pathology. Interestingly, people with MCI without carer help for transportation were also significantly less likely to incur annual PBS costs, a result consistent across both the unbalanced sample and attrition-adjusted estimates.

**Table 5:** Subgroup analysis through interactions with MCI – Part 2 coefficients [Reference: normal cognition].

	*(D1) Unbalanced sample GEE*			*(D3) IPW-weighted GEE*		
	Coeff.	S.E	P	Coeff.	S.E	P
*All MBS*						
MCI Male	0.061	0.069	0.377	0.079	0.094	0.403
MCI Female	−0.033	0.077	0.667	−0.043	0.081	0.594
MCI, Past occupation: Managerial/Professional)	0.111*	0.065	0.088	0.114	0.090	0.205
MCI, Past occupation: Non-Managerial/Professional)	−0.118	0.082	0.153	−0.106	0.082	0.197
MCI, amnestic	0.021	0.063	0.734	0.039	0.078	0.614
MCI, non-amnestic	0.003	0.070	0.971	−0.010	0.084	0.902
MCI, lives alone	0.062	0.084	0.462	0.057	0.083	0.496
MCI, does not live alone	−0.010	0.064	0.881	0.0002	0.0871	0.9980
MCI, carer helps with making medical decisions	0.108	0.087	0.216	0.100	0.115	0.384
MCI, carer does not help with making medical decisions	−0.019	0.062	0.764	−0.006	0.076	0.936
MCI, carer helps with transport	0.092	0.084	0.272	0.119	0.110	0.281
MCI, carer does not help with transport	−0.019	0.063	0.767	−0.025	0.074	0.736
*All PBS*						
MCI Male	−0.124	0.088	0.159	−0.117	0.107	0.277
MCI Female	0.168	0.111	0.131	0.166	0.132	0.207
S						
MCI, Past occupation: Managerial/Professional)	−0.072	0.094	0.442	−0.064	0.124	0.607
MCI, Past occupation: Non-Managerial/Professional)	0.033	0.097	0.731	0.030	0.125	0.814
MCI, amnestic	−0.081	0.087	0.354	−0.068	0.108	0.530
MCI, non-amnestic	0.019	0.089	0.833	0.010	0.103	0.923
MCI, lives alone	−0.115	0.107	0.283	−0.134	0.117	0.253
MCI, does not live alone	0.032	0.089	0.719	0.055	0.110	0.619
MCI, carer helps with making medical decisions	−0.416***	0.119	0.000	−0.423**	0.173	0.014
MCI, carer does not help with making medical decisions	0.152	0.090	0.093	0.168	0.119	0.158
MCI, carer helps with transport	0.148	0.108	0.170	0.148	0.173	0.393
MCI, carer does not help with transport	−0.097	0.085	0.255	−0.089	0.086	0.304
*Professional attendances*						
MCI Male	0.065	0.058	0.266	0.076	0.069	0.265
MCI Female	−0.059	0.060	0.333	−0.062	0.064	0.335
MCI, Past occupation: Managerial/Professional)	0.040	0.057	0.479	0.040	0.064	0.534
MCI, Past occupation: Non-Managerial/Professional)	−0.039	0.062	0.524	−0.029	0.070	0.676
MCI, amnestic	−0.007	0.053	0.887	0.003	0.055	0.953
MCI, non-amnestic	0.013	0.056	0.823	0.008	0.055	0.884
MCI, lives alone	0.037	0.065	0.564	0.029	0.067	0.661
MCI, does not live alone	−0.026	0.054	0.636	−0.013	0.059	0.826
MCI, carer helps with making medical decisions	0.035	0.077	0.646	0.033	0.080	0.680
MCI, carer does not help with making medical decisions	−0.010	0.049	0.844	−0.003	0.055	0.957
MCI, carer helps with transport	0.000	0.070	0.998	0.011	0.084	0.896
MCI, carer does not help with transport	0.003	0.051	0.960	0.003	0.052	0.955
*Diagnostic imaging*						
MCI Male	−0.134	0.099	0.177	−0.121	0.106	0.255
MCI Female	−0.094	0.111	0.398	−0.135	0.120	0.260
MCI (Past occupation: Managerial/Professional)	−0.084	0.098	0.389	−0.088	0.097	0.366
MCI (Past occupation: Non-Managerial/Professional)	−0.159	0.113	0.161	−0.176	0.129	0.172
MCI amnestic	−0.082	0.090	0.363	−0.079	0.101	0.434
MCI non-amnestic	−0.158	0.106	0.137	−0.187*	0.102	0.065
MCI and lives alone	−0.179*	0.094	0.057	−0.188*	0.097	0.053
MCI and does not live alone	−0.005	0.117	0.967	−0.025	0.124	0.837
MCI and carer helps with making medical decisions	−0.099	0.126	0.432	−0.122	0.114	0.283
MCI and carer does not help with making medical decisions	−0.134	0.089	0.133	−0.135	0.099	0.175
MCI and carer helps with transport	−0.020	0.115	0.860	−0.021	0.139	0.880
MCI and carer does not help with transport	−0.175*	0.093	0.061	−0.192**	0.085	0.024
*Pathology*						
MCI Male	0.103	0.094	0.276	0.123	0.122	0.316
MCI Female	−0.093	0.111	0.402	−0.150	0.120	0.212
MCI (Past occupation: Managerial/Professional)	0.061	0.095	0.522	0.043	0.114	0.707
MCI (Past occupation: Non-Managerial/Professional)	−0.041	0.107	0.703	−0.045	0.133	0.738
MCI amnestic	0.049	0.087	0.572	0.040	0.105	0.704
MCI non-amnestic	−0.034	0.099	0.734	−0.052	0.108	0.634
MCI and lives alone	−0.058	0.092	0.529	0.080	0.122	0.513
MCI and does not live alone	0.135	0.112	0.225	−0.042	0.109	0.698
MCI and carer helps with making medical decisions	0.022	0.122	0.858	0.035	0.144	0.809
MCI and carer does not help with making medical decisions	0.008	0.086	0.922	−0.012	0.100	0.908
MCI and carer helps with transport	0.007	0.115	0.954	0.004	0.143	0.976
MCI and carer does not help with transport	0.020	0.088	0.823	0.0001	0.100	1.000
*Nervous system drugs*						
MCI Male	0.371**	0.163	0.023	0.344	0.240	0.151
MCI Female	0.225	0.174	0.196	0.199	0.214	0.354
MCI (Past occupation: Managerial/Professional)	0.230	0.156	0.141	0.184	0.181	0.311
MCI (Past occupation: Non-Managerial/Professional)	0.466**	0.198	0.019	0.400	0.251	0.111
MCI amnestic	0.309**	0.147	0.036	0.286	0.193	0.137
MCI non-amnestic	0.291*	0.172	0.091	0.256	0.212	0.228
MCI and lives alone	0.464**	0.230	0.044	0.398*	0.204	0.051
MCI and does not live alone	0.286*	0.147	0.051	0.242	0.229	0.291
MCI and carer helps with making medical decisions	0.275	0.259	0.288	0.299	0.389	0.442
MCI and carer does not help with making medical decisions	0.286	0.215	0.184	0.261	0.268	0.330
MCI and carer helps with transport	0.249	0.158	0.116	0.235	0.230	0.305
MCI and carer does not help with transport	0.285*	0.165	0.085	0.285	0.201	0.156

* Full modelling results for all covariates available from the authors. P < 0.1*, p < 0.05**, p < 0.01***.

Females with MCI were significantly less likely than males with MCI to incur annual diagnostic imaging and pathology costs. People with MCI in non-managerial and professional past occupations were also significantly less likely to incur annual MBS costs than cognitively normal individuals (with no significant differences for those in managerial/professional past occupations).

For the second model part (annual amount of costs incurred), there were some differences in statistical significance across the unbalanced sample and attrition-adjusted estimates. The results that were consistent across these two sets included significantly reduced annual diagnostic imaging costs incurred by individuals with MCI who lived alone or whose carer does not help with transport relative to cognitively normal individuals. Interestingly, individuals with MCI whose carers helped them with medical decisions incurred significantly less annual PBS costs than cognitively normal individuals, while individuals with MCI who lived alone incurred significantly more nervous system drugs costs than cognitively normal individuals.

## 4 Discussion

We found that people with MCI had slightly lower predicted annual costs for diagnostic imaging, pathology, and professional attendances than cognitively normal individuals. Conversely, people with severe impairment, as indicated by a dementia diagnosis, had substantially higher predicted annual professional attendances and nervous system drugs costs relative to cognitively normal individuals in the year following diagnosis.

We found that people diagnosed with MCI in MAS were significantly *less likely* to incur medical costs, including pathology and diagnostic imaging, than cognitively normal individuals, with this effect concentrated in people with non-amnestic MCI, people with MCI who lived alone and those with little carer support with transport and making medical decisions. Further subgroup analyses found that individuals with MCI who lived alone or did not have carer help with transport also incurred significantly less annual cost amounts for diagnostic imaging. These results align with US findings on potential outpatient service gaps for undiagnosed people with cognitive impairment living in the community [4 6 17 21].

Our results suggest that living arrangements, carer support and non-amnestic symptoms are potential moderators of using medical care for people with MCI. Within the MAS, since MCI incidences not shared with participants, participants with MCI were also unaware of their meeting MCI diagnostic criteria, and underinformed about best practices for managing potential disease progression. A recent study from the UK reported similar findings for other health care types, noting that people with milder cognitive impairment had a significantly lower uptake of allied health services, dentist appointments and sight checks, indicating potential service gaps may extend to other care types beyond medical services for this population (MacLeod et al., 2021).

Recent research (Woodward et al., 2022) summarises expert-informed recommendations for detecting, assessing and managing MCI in primary care, which could be promoted among Australian healthcare providers. These include regular reviews for patients with MCI by GPs or specialists, initiating triggers for early reviews after diagnosis, training and support for GPs, increasing awareness about lifestyle interventions for MCI, management of mood-related and other chronic diseases in addition to medication management and better tailoring of care to patients. Increasing timely diagnoses of MCI and early dementia using cognitive screening tests administered by GPs has also been found to be cost-effective, in terms of delivering improved quality of life for patients and generating savings across the health care, social care and informal care sectors (Tong et al., 2017).

More research is warranted on potential gaps in the use of medical care for people with MCI, and if these are deemed to exist, future health services may need to be better targeted to reach and diagnose this group, as well as to support these individuals. This is particularly important given that a lack of use of primary care and follow-ups for MCI management and counselling (Chen et al., 2021) may translate into more costly hospital inpatient utilisation (Caspi et al., 2009), with the cost impacts likely to accelerate as the population in developed countries ages rapidly.

Home services (Burgdorf et al., 2022) and the ongoing expansion of telehealth (Fisk & Livingstone, 2020) hold promise in this area and may help increase health care access, use and diagnoses for under-serviced older adults experiencing cognitive impairment including those living alone, those with inadequate carer support or those with transportation difficulties.

### 4.1 Limitations

While this study offers insights on health care use and patterns for people with MCI and dementia in the Australian community, we acknowledge that this research is subject to several limitations.

The first is that we estimated statistical associations between cognitive performance and diagnoses, and medical and pharmaceutical use and costs in the year following, but these should not be taken as causal estimates. In particular, there may be potential endogeneity between cognition and health care if there is reverse causality between health care spending and cognition (e.g. health care spending in one year resulting in improved cognitive scores or reversion of MCI diagnoses in the next MAS wave). Nonetheless, the causal link between early intervention (including medical care and pharmaceuticals) and slowing progression to dementia or reversing cognitive decline is an area of ongoing research, with no conclusive findings (Dooley et al., 2020).

Secondly, while study participants were randomly recruited, the MAS sample was drawn from two relatively high socioeconomic status, adjacent geographic regions. The sample was also relatively homogeneous in terms of ethnicity (with 93% of participants born in Australia/NZ and Europe/UK) (Sachdev et al., 2010). While there is unlikely to be substantial variations in socioeconomic status *within* the participant sample (and we controlled for residual differences using years of education and past occupation as covariates), we note that drawing the sample from these regions and relatively homogenous ethnicity group may limit the generalisability of findings to the broader population.

While we analysed MBS and PBS expenses, most of which are funded by the Government and covered by subsidy arrangements for older people (Appendix A), individuals may still face varying levels of out-of-pocket costs for specific MBS items such as specialist attendances. Hence, cost barriers may be more pronounced across the general older Australian community. Since our overall study finding is of potential service gaps for people with MCI from relatively advantaged regions, we posit that these findings may be even more pronounced for individuals with undiagnosed MCI in lower socioeconomic status regions due to cost barriers, or those from more culturally and linguistically diverse backgrounds facing language barriers.

The MAS panel used in our estimations was subject to high attrition by the fourth wave due to drop-out, death and withdrawal which is typical of panel surveys with participants of older ages and in more frail health. To adjust our estimates for attrition, we used an IPW approach based on a ‘missing at random’ assumption, which assumes that attrition is ignorable after adjusting for covariates associated with drop-out during the survey and reweighting the sample based on response probability. A ‘missing at random’ approach assumes that drop-out is likely to depend on observed (mostly health related) covariates, and that missingness is not systematically related to unobserved data (Carreras et al., 2021). We acknowledge that the treatment of missing data in this study is limited by the validity of this assumption.

The robustness of our estimation results is also contingent on the assumptions of the modelling approaches we have chosen in this study. One assumption in applying random effects estimation (compared to fixed effects) to pooled panel data is that of the individual-constant unobserved effect being uncorrelated with covariates in each time period. We acknowledge this is a strong assumption. Nonetheless, random effects estimation was deemed more appropriate to our setting and for our overall study objective, where we did not desire to remove the individual-specific, time-invariant contribution of genes and hereditary factors related to cognitive impairment.

As discussed in more detail in Appendix Section A, while we estimated total PBS costs as the sum of patient contributions and government benefits payable (based on listed PBS prices), this may not reflect the actual cost for some newly listed drugs on the PBS since actual amounts paid by Government may be determined by confidential pricing or risk sharing arrangements.

Lastly, this study focused on medical and pharmaceutical costs associated with diagnosed and undiagnosed cognitive impairment. We acknowledge that there are many other economic and health care cost components, such as hospital costs, aged care (including home nursing and nursing homes) and informal care, that are provided to people with severe cognitive impairment [8 57] and these are also important to estimate and quantify in updated future studies on the older population. However, our specific focus within this study was to explore medical care and pharmaceutical use patterns for older people in the Australian community, to better inform primary care policy related to the diagnosis and management of MCI and encourage earlier, cost-effective interventions.

## 5 Conclusion

Our study is the first to identify potential gaps in medical service use for people with undiagnosed MCI - and especially non-amnestic MCI - living in the Australian community, which fares relatively better than the US in terms of cost and time barriers to accessing health care (Commonwealth Fund, 2021). We argue these results provide evidence for the importance of adequate primary care screening for MCI, communication of diagnoses, increasing awareness of non-amnestic symptoms and encouraging help-seeking behaviours and community-driven support for individuals with MCI, particularly those living alone and with inadequate carer support.

## Ethics statement

All participants and informants provided written consent to participate in the Sydney Memory and Ageing study, including consent to access and link their health records through Services Australia. The consent form and process was approved by the University of New South Wales Human Ethics Review Committee (HC 05037, 09382, 14327, 190962).

## Funding

The Sydney Memory and Ageing Study (MAS) was funded by three National 10.13039/100018696Health & 10.13039/501100000265Medical Research Council (10.13039/501100000925NHMRC) Program Grants (ID350833, ID568969, and APP1093083) awarded to 10.13039/501100016226HB and PSS.

## CRediT authorship contribution statement

**Anam Bilgrami:** Conceptualization, Formal analysis, Methodology, Writing – original draft, Writing – review & editing. **Mona Aghdaee:** Conceptualization, Formal analysis, Methodology, Writing – original draft, Writing – review & editing. **Yuanyuan Gu:** Conceptualization, Formal analysis, Methodology, Supervision, Writing – review & editing. **Henry Cutler:** Conceptualization, Methodology, Supervision, Writing – review & editing. **Katya Numbers:** Conceptualization, Data curation, Investigation, Resources, Writing – review & editing. **Nicole A. Kochan:** Data curation, Resources. **Perminder S. Sachdev:** Funding acquisition, Resources, Supervision, Writing – review & editing. **Henry Brodaty:** Conceptualization, Funding acquisition, Methodology, Resources, Supervision, Writing – review & editing.

## Declaration of competing interest

HB is or has been an advisory board member or consultant to Biogen, Nutricia, Roche and Skin2Neuron. He is a Medical/Clinical Advisory Board member for Montefiore Homes and Cranbrook Care. PSS served on the advisory committees of Biogen Australia and Roche Australia in 2020 and 2021.

## Data Availability

The data that has been used is confidential.

## Footnotes

1 These characteristics included the global z-score and the set of covariates in Section 2.1.4 (excluding death during the survey which itself is a form of drop-out). We also included additional health history variables such as recent self-reported hospital admission, history of kidney disease and history of depression, which have been found to be significantly associated with MAS attrition in previous research [48 49].

2 Namely, lower z-scores, higher age, lower years of education, more comorbidities, higher alcohol consumption, lower physical activity, lower AQOL-6D scores, hospital admission around Wave 1 and a history of kidney disease were all associated with a greater probability of dropping out of the MAS.

3 For the first modelling part, the IPW probability weights were applied to a pooled rather than random effects logit model, since probability weights can only be applied to the population-averaged estimation, rather than the panel (random) estimation.
