# Supplementary material for: Patterns in health care use and intensity for diagnosed and undiagnosed cognitive impairment in the older australian community: Implications for primary care management
Source: SSM Popul Health. 2024 Jul 2;27:101693. doi: 10.1016/j.ssmph.2024.101693 (PMC11838139; doi:10.1016/j.ssmph.2024.101693)
Supplement: Multimedia component 1 [file mmc1.docx]

# APPENDICES

**Appendix A: Information on the Medicare Benefits Schedule (MBS) and Pharmaceutical Benefits Scheme (PBS)**

The MBS is a listing of medical treatments subsidised by the Australian Government up to a pre-determined benefit amount. Generally, the types of services on the MBS include consultations with doctors (including general practitioners and specialists), procedural and therapeutic services (including surgical) and diagnostic services. While the MBS may be used to cover the costs of medical services provided to private patients in public or private hospitals, it cannot be billed for costs incurred by public patients in hospital, with these covered by the Australian Government, and state and territory governments, through activity-based funding arrangements. For this reason, we focus only on medical costs incurred outside of the hospital setting within this study. The MBS does not cover theatre fees and accommodation costs incurred by private patients, dental examinations and treatments, ambulance services, home nursing, hearing aids and appliances, prostheses, acupuncture and non-clinically necessary services. Most Medicare services (83 per cent) are bulk-billed (Department of Health, 2022a), where the provider charges exactly the government-paid benefit and there is no patient co-payment.

Under the PBS, the Australian Government subsidises the cost of listed medicines, with most of these dispensed by pharmacists, and used by patients at home. For PBS listed medicines, individuals pay a proportion (co-payment) of the price for a medicine and the Government pays the rest. The PBS subsidises prescriptions for most medical conditions, but does not cover over-the-counter medicines, private prescriptions (i.e. medicines not available in Australia or not listed on the PBS) and medicines provided to inpatients (Department of Health, 2022b). Medications listed on the PBS cannot be advertised in Australia.

Eligible older Australians receive further reductions in co-payments if they hold a Pensioner Concession Card (for age pension recipients) (Services Australia, 2021a), Commonwealth Seniors Health Card (for self-funded retirees) (Services Australia, 2021b) or reach concessional status, allowing them to access cheaper PBS medicines at a capped rate (currently AUD$6.80). They also have lower thresholds for reaching the MBS and PBS ‘Safety Net’ concessional status (Department of Health, 2022c; Services Australia, 2022) which are annual limits applying out-of-pocket MBS and PBS expenditure, after which they receive further discounts or waiving of MBS co-payments and free PBS medicines. Most older Australians have access to these benefits, given that over two-thirds alone receive the age pension (Australian Institute of Health and Welfare, 2021).

In this study, while we estimated total PBS costs as the sum of patient contributions and government benefits payable (based on listed PBS prices), this may not reflect the actual cost for some *newly listed* drugs on the PBS since actual amounts paid by Government may be determined by confidential Special Pricing Arrangements (SPAs) or Risk Sharing Arrangements (RSAs) (Department of Health, 2017a). SPAs may be entered into to account for differences between published and ‘cost-effective’ prices, while RSAs may be needed to address risks such as uncertainty in estimating costs to the PBS, varying cost-effectiveness across volumes of use, data monitoring and usage uncertainty (Department of Health, 2017b).

Appendix A References:

Australian Institute of Health and Welfare. *Older Australians - Income and Finances. Secondary Older Australians - Income and Finances* 2021. https://www.aihw.gov.au/reports/older-people/older-australians/contents/income-and-finances#Income%20during%20retirement.

Department of Health. *Total Medicare Statistics, 2021-22 Jul-Dec YTD. Secondary Total Medicare Statistics, 2021-22 Jul-Dec YTD* 2022a. https://www1.health.gov.au/internet/main/publishing.nsf/Content/32CC6EB4BCC0BB1CCA257BF0001FEB92/$File/Medicare%20year%20to%20date%20dashboard.pdf.

Department of Health. *About the PBS. Secondary About the PBS* 2022b. <https://www.pbs.gov.au/info/about-the-pbs#What_medicines_does_the_government_subsidise>.

Department of Health. *MBS Online: Medicare Safety Net Arrangement - 1 January 2021. Secondary MBS* *Online: Medicare Safety Net Arrangement* – 2022c. <http://www.mbsonline.gov.au/internet/mbsonline/publishing.nsf/Content/Factsheet-EMSN1Jan2021>.

Department of Health. *The Pharmaceutical Benefits Scheme - The Deed of Agreement Process. Secondary The Pharmaceutical Benefits Scheme - The Deed of Agreement Process* 2017a. https://www.pbs.gov.au/info/industry/listing/elements/deeds-agreement/c-deed-of-agreement.

Department of Health. *The Pharmaceutical Benefits Scheme - Background - What are Deeds of Agreement? Secondary The Pharmaceutical Benefits Scheme - Background - What are Deeds of Agreement?* 2017b. https://www.pbs.gov.au/pbs/industry/listing/elements/deeds-agreement/b-background.

Services Australia. *Pensioner Concession Card. Secondary Pensioner Concession Card* 2021a. https://www.servicesaustralia.gov.au/pensioner-concession-card.

Services Australia. *Commonwealth Seniors Health Card. Secondary Commonwealth Seniors Health Card* 2021b. https://www.servicesaustralia.gov.au/commonwealth-seniors-health-card.

Services Australia. *PBS Safety Net thresholds. Secondary PBS Safety Net thresholds* 2022. https://www.servicesaustralia.gov.au/pbs-safety-net-thresholds?context=22016.

**Appendix B: Supplementary tables and figures**

**Table B1: Neuropsychological test battery administered in the Sydney Memory and Ageing Study**

| ***Cognitive domain*** | ***Test*** |
| --- | --- |
| Premorbid intelligence | Premorbid intelligence National Adult Reading Test (NART) |
|  |  |
| Attention/processing speed | Attention/processing speed Digit Symbol-Coding, Trail Making Test A |
|  |  |
| Memory | Logical Memory Story A delayed recall, Rey Auditory Verbal Learning Test (RAVLT), RAVLT total learning; trials 1–5, RAVLT short-term delayed recall; trial 6, RAVLT long-term delayed recall; trial 7, Benton Visual Retention Test recognition |
|  |  |
| Language | Boston Naming Test (30 items), Semantic Fluency (Animals) |
|  |  |
| Visuo-spatial | Block Design |
|  |  |
| Executive function | Controlled Oral Word Association Test (FAS), Trail Making Test B |
|  |  |
| Fine Motor | Grooved Pegboard Test |

* Raw test scores were standardised against a “healthy” wave 1 reference group, so that the mean and standard deviations were equal to zero and one, resulting in “quasi z-scores”, that were adjusted for participant age and education. These z-scores represent an ‘objective’ measure of cognitive impairment across different domains.

† Full test details, test sources and normative data/demographic adjustments available in Sachdev, P. S., Brodaty, H., Reppermund et al. (2010). The Sydney Memory and Ageing Study (MAS): methodology and baseline medical and neuropsychiatric characteristics of an elderly epidemiological non-demented cohort of Australians aged 70–90 years. International psychogeriatrics, 22(8), 1248-1264.

**Figure B1: Cost distributions for Sydney MAS participants, waves 1-4^(a)^**


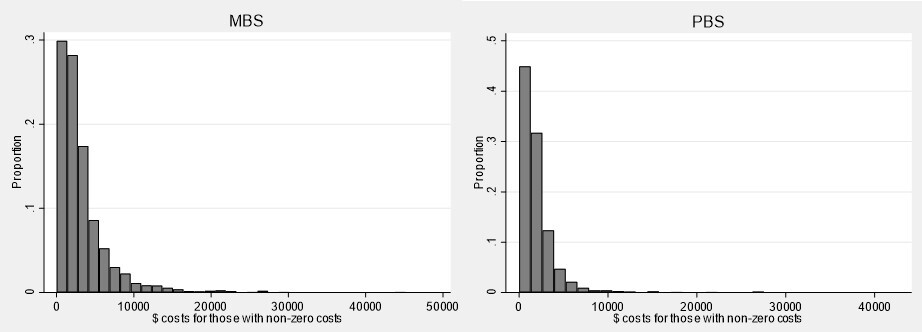


(a) ‘Proportion’ on vertical axes refers to proportion of all participants in the sample

**Figure B2: Sydney Memory and Ageing Study – overlap between z-score and MCI diagnoses^(a)^**

1. ‘Proportion’ on vertical axis refers to ‘proportion of all participants in the sample’

**Figure B3: Sydney Memory and Ageing Study – overlap between z-score and dementia diagnoses^(a)^**

(a) ‘Proportion’ on vertical axis refers to ‘proportion of all participants in the sample’

**Figure B4: Part 1 (random effects logit) marginal effects and confidence intervals by z-score levels**

**Table B2: Part one modelling coefficients**

| ***(A) Z-score*** | | | | | | | | | | | | | | | | | |
| --- | --- | --- | --- | --- | --- | --- | --- | --- | --- | --- | --- | --- | --- | --- | --- | --- | --- |
|  | ***(A1) Unbalanced sample (random effects logit)*** | | | ***(A2) Balanced sample (random effects logit)*** | | | | ***(A3) IPW-weighted***  ***(pooled logit)*** | | | | | | | | | |
|  | ***Coeff.*** | ***S.E*** | ***P*** | ***Coeff.*** | | ***S.E*** | ***P*** | ***Coeff.*** | ***S.E*** | | | | ***P*** | | | | |
| **All MBS:** |  |  |  |  | |  |  |  | | | | | | | | | |
| z-score | **0.746^***^** | **0.216** | **0.001** | **0.632^**^** | | **0.309** | **0.041** | **0.228^***^** | | **0.082** | | | **0.006** | | | | |
| z-score squared | **0.173^**^** | **0.078** | **0.027** | 0.166 | | 0.108 | 0.125 | **0.066^**^** | | **0.029** | | | **0.022** | | | | |
| *N (individuals)* | *3,010 (1,009 individuals)* | | | *2,512 (703 individuals)* | | | | *2,911 (965 individuals)* | | | | | | | | | |
|  |  | | |  | |  |  |  | | | | | | | | | |
| **Prof.attend. (MBS):** |  |  |  |  | |  |  |  | |  | | |  | | | | |
| z-score | **0.302^***^** | **0.085** | **0.000** | **0.264^**^** | | **0.105** | **0.012** | **0.178^***^** | | **0.051** | | | **0.001** | | | | |
| z-score squared | **0.124^***^** | **0.031** | **0.000** | **0.118^***^** | | **0.043** | **0.006** | **0.070^***^** | | **0.019** | | | **0.000** | | | | |
| *N (individuals)* | *3,010 (1,009 individuals)* | | | *2,512 (703 individuals)* | | | | *2,911 (965 individuals)* | | | | | | | | | |
|  |  |  |  |  | |  |  |  | | | | | | | | | |
| **Diag. imag. (MBS):** |  |  |  |  | |  |  |  | |  | | |  | | | | |
| z-score | **0.166^**^** | **0.066** | **0.011** | 0.117 | | 0.073 | 0.111 | **0.144^***^** | | **0.050** | | | **0.004** | | | | |
| z-score squared | **0.057^***^** | **0.020** | **0.005** | **0.070^***^** | | **0.025** | **0.005** | **0.056^***^** | | **0.011** | | | **0.001** | | | | |
| *N (individuals)* | *3,010 (1,009 individuals)* | | | *2,512 (703 individuals)* | | | | *2,911 (965 individuals)* | | | | | | | | | |
|  |  |  |  |  | |  |  |  | | | | | | | | | |
| **Pathology (MBS):** |  |  |  |  | |  |  |  |  | | | |  | | | | |
| z-score | **0.221^***^** | **0.073** | **0.002** | **0.204** | | **0.084** | **0.015** | **0.150** | **0.050** | | | | **0.003** | | | | |
| z-score squared | **0.090^***^** | **0.026** | **0.001** | **0.075** | | **0.029** | **0.011** | **0.058** | **0.019** | | | | **0.003** | | | | |
| *N (individuals)* | *3,010 (1,009 individuals)* | | | *2,512 (703 individuals)* | | | | *2,911 (965 individuals)* | | | | | | | | | |
|  |  |  |  |  | |  |  |  | | | | | | | | | |
| **All PBS:** |  |  |  |  | |  |  |  |  | | | |  | | | | |
| z-score | **0.342^**^** | **0.156** | **0.028** | 0.271 | | 0.188 | 0.149 | **0.121^*^** | **0.070** | | | | **0.085** | | | | |
| z-score squared | 0.039 | 0.045 | 0.388 | 0.022 | | 0.053 | 0.675 | 0.026 | 0.023 | | | | 0.248 | | | | |
| *N (individuals)* | *3,010 (1,009 individuals)* | | | *2,512 (703 individuals)* | | | | *2,911 (965 individuals)* | | | | | | | | | |
|  |  |  |  |  | |  |  |  | | | | | | | | | |
| **Nerv.sys. (PBS)** |  |  |  |  | |  |  |  |  | | | |  | | | | |
| z-score | **-0.231^**^** | **0.108** | **0.032** | **-0.252^**^** | | **0.123** | **0.041** | **-0.118^**^** | **0.047** | | | | **0.012** | | | | |
| z-score squared | 0.002 | 0.032 | 0.939 | -0.006 | | 0.036 | 0.875 | 0.004 | 0.016 | | | | 0.819 | | | | |
| *N (individuals)* | *3,010 (1,009 individuals)* | | | *2,512 (703 individuals)* | | | | *2,911 (965 individuals)* | | | | | | | | | |
| ***(B) Diagnoses [Reference category: normal cognition]*** | | | | | | | | | | | | | | | | | |
|  | ***(B1) Unbalanced sample (random effects logit)*** | | | ***(B2) Balanced sample (random effects logit)*** | | | | ***(B3) IPW-weighted (pooled logit)*** | | | | | | | | | |
|  | ***Coeff.*** | ***S.E*** | ***P*** | ***Coeff.*** | | ***S.E*** | ***P*** | ***Coeff.*** | | | ***S.E*** | | | | | | ***P*** |
| **All MBS** |  |  |  |  | |  |  |  | | | | | | | | | |
| MCI | **-0.562^*^** | **0.310** | **0.069** | -0.518 | | 0.378 | 0.170 | **-0.225^*^** | | | **0.129** | | | | | | **0.081** |
| Dementia | 0.328 | 0.762 | 0.667 | 0.647 | | 0.837 | 0.439 | 0.444 | | | 0.373 | | | | | | 0.234 |
| *N (individuals)* | *2,676 (955 individuals)* | | | *2,262 (686 individuals)* | | | | *2,585 (915 individuals)* | | | | | | | | | |
|  |  |  |  |  | |  |  |  | | | | | | | | | |
| **Prof. attend (MBS)** |  |  |  |  | |  |  |  | | | | | | | | | |
| MCI | -0.216 | 0.151 | 0.153 | -0.217 | | 0.188 | 0.249 | -0.141 | | | 0.098 | | | | | | 0.151 |
| Dementia | **1.358^***^** | **0.523** | **0.009** | **1.264^**^** | | **0.624** | **0.043** | **1.021^***^** | | | **0.348** | | | | | | **0.003** |
| *N (individuals)* | *2,676 (955 individuals)* | | | *2,262 (686 individuals)* | | | | *2,585 (915 individuals)* | | | | | | | | | |
|  |  |  |  |  | |  |  |  | | | | | | | | | |
| **Diag. imag. (MBS)** |  |  |  |  | |  |  |  | | | | | | | | | |
| MCI | **-0.229^*^** | **0.121** | **0.060** | -0.188 | | 0.136 | 0.165 | **-0.162^*^** | | | **0.096** | | | | | **0.089** | |
| Dementia | 0.391 | 0.348 | 0.262 | 0.610 | | 0.394 | 0.122 | 0.432 | | | 0.289 | | | | | 0.135 | |
| *N (individuals)* | *2,676 (955 individuals)* | | | *2,262 (686 individuals)* | | | | *2,585 (915 individuals)* | | | | | | | | | |
|  |  |  |  |  | |  |  |  | | | | | | | | | |
| **Pathology (MBS)** |  |  |  |  | |  |  |  | | | | | | | | | |
| MCI | **-0.228^*^** | **0.129** | **0.078** | -0.211 | | 0.148 | 0.154 | **-0.166^*^** | | | **0.096** | | | | | **0.084** | |
| Dementia | **1.559^***^** | **0.458** | **0.001** | **1.294^***^** | | **0.484** | **0.007** | **1.195^***^** | | | **0.355** | | | | | **0.001** | |
| *N (individuals)* | *2,676 (955 individuals)* | | | *2,262 (686 individuals)* | | | | *2,585 (915 individuals)* | | | | | | | | | |
|  |  |  |  |  | |  |  |  | | | | | | | | | |
| **All PBS** |  |  |  |  | |  |  |  | | | | | | | | | |
| MCI | -0.194 | 0.244 | 0.426 | -0.239 | 0.273 | | 0.380 | -0.199 | | | 0.123 | | | | 0.105 | | |
| Dementia | 0.031 | 0.713 | 0.965 | -0.000 | 0.732 | | 1.000 | 0.131 | | | 0.349 | | | | 0.708 | | |
| *N (individuals)* | *2,676 (955 individuals)* | | | *2,262 (686 individuals)* | | | | *2,585 (915 individuals)* | | | | | | | | | |
|  |  |  |  |  | |  |  |  | | | | | | | | | |
| **Nerv. syst. (PBS)** |  |  |  |  | |  |  |  | | | | | | | | | |
| MCI | -0.124 | 0.175 | 0.479 | -0.130 | | 0.192 | 0.499 | 0.053 | | | 0.090 | | | | 0.551 | | |
| Dementia | **1.155^**^** | **0.505** | **0.022** | **1.227^**^** | | **0.549** | **0.025** | **0.923^***^** | | | | **0.275** | | **0.001** | | | |
| *N (individuals)* | *2,676 (955 individuals)* | | | *2,262 (686 individuals)* | | | | *2,585 (915 individuals)* | | | | | | | | | |

* Full modelling results for all covariates available from the authors. **p<0.1*, p<0.05**, p<0.01*****

**Table B3: Part two modelling coefficients**

| ***C) Z-score*** | | | | | | | | | |
| --- | --- | --- | --- | --- | --- | --- | --- | --- | --- |
|  | ***(C1) Unbalanced sample GEE*** | | | ***(C2) Balanced sample GEE*** | | | ***(C3) IPW-weighted GEE*** | | |
|  | ***Coeff.*** | ***S.E*** | ***P*** | ***Coeff.*** | ***S.E*** | ***P*** | ***Coeff.*** | ***S.E*** | ***P*** |
| **All MBS:** |  |  |  |  |  |  |  |  |  |
| z-score | -0.038 | 0.029 | 0.182 | -0.038 | 0.031 | 0.224 | -0.027 | 0.033 | 0.400 |
| z-score squared | -0.010 | 0.010 | 0.320 | -0.006 | 0.010 | 0.560 | -0.006 | 0.011 | 0.558 |
| *N (individuals)* | *2,596 (885 individuals)* | | | *2,245 (655 individuals)* | | | *2,508 (844 individuals)* | | |
|  |  | | |  | | |  | | |
| **Prof. att. (MBS)** |  | | |  | | |  | | |
| z-score | -0.060 | 0.023 | 0.010 | -0.063 | 0.025 | 0.010 | -0.053 | 0.025 | 0.035 |
| z-score squared | -0.002 | 0.006 | 0.790 | 0.003 | 0.006 | 0.659 | 0.003 | 0.007 | 0.716 |
| *N (individuals)* | *1,794 (734 individuals)* | | | *1,661 (624 individuals)* | | | *1,707 (693 individuals)* | | |
|  |  |  |  |  |  |  |  |  |  |
| **Diag. imag. (MBS):** |  |  |  |  |  |  |  |  |  |
| z-score | 0.052 | 0.037 | 0.161 | 0.045 | 0.039 | 0.244 | 0.067 | 0.054 | 0.214 |
| z-score squared | 0.005 | 0.012 | 0.674 | 0.007 | 0.012 | 0.564 | 0.008 | 0.016 | 0.616 |
| *N (individuals)* | *1,481 (688 individuals)* | | | *1,372 (595 individuals)* | | | *1,409 (652 individuals)* | | |
|  |  | | |  | | |  | | |
| **Pathology (MBS):** |  |  |  |  |  |  |  |  |  |
| z-score | **-0.080^*^** | **0.041** | **0.048** | **-0.082^*^** | **0.043** | **0.056** | **-0.070^*^** | **0.040** | **0.082** |
| z-score squared | -0.023 | 0.014 | 0.103 | -0.020 | 0.015 | 0.171 | -0.019 | 0.012 | 0.123 |
| *N (individuals)* | *1,610 (709 individuals)* | | | *1,487 (606 individuals)* | | | *1,532 (672 individuals)* | | |
|  |  | | |  | | |  | | |
| **All PBS:** |  |  |  |  |  |  |  |  |  |
| z-score | -0.063 | 0.041 | 0.130 | **-0.081^*^** | **0.046** | **0.080** | -0.052 | 0.067 | 0.437 |
| z-score squared | -0.003 | 0.009 | 0.729 | -0.005 | 0.010 | 0.633 | 0.006 | 0.012 | 0.652 |
| *N (individuals)* | *2,547 (887 individuals)* | | | *2,191 (654 individuals)* | | | *2,460 (847 individuals)* | | |
|  |  | | |  | | |  | | |
| **Nerv.sys. (PBS)** |  |  |  |  |  |  |  |  |  |
| z-score | **-0.571^***^** | **0.073** | **0.000** | **-0.415^***^** | **0.083** | **0.000** | **-0.499^***^** | **0.122** | **0.000** |
| z-score squared | **-0.035^***^** | **0.012** | **0.004** | -0.017 | 0.016 | 0.299 | 0.009 | 0.019 | 0.641 |
| *N (individuals)* | *1,291 (589 individuals)* | | | *1,093 (445 individuals)* | | | *1,238 (562 individuals)* | | |
| ***(D) Diagnoses [Reference category: normal cognition]*** | | | | | | | | | |
|  | ***(D1) Unbalanced sample GEE*** | | | ***(D2) Balanced sample GEE*** | | | ***(D3) IPW-weighted***  ***GEE*** | | |
|  | ***Coeff.*** | ***S.E*** | ***P*** | ***Coeff.*** | ***S.E*** | ***P*** | ***Coeff.*** | ***S.E*** | ***P*** |
| **All MBS** |  |  |  |  |  |  |  |  |  |
| MCI | 0.018 | 0.052 | 0.727 | 0.030 | 0.057 | 0.601 | 0.023 | 0.063 | 0.715 |
| Dementia | -0.027 | 0.119 | 0.817 | 0.008 | 0.126 | 0.949 | -0.019 | 0.156 | 0.902 |
| *N (individuals)* | *2,307 (835 individuals)* | | | *2,011 (634 individuals)* | | | *2,226 (799 individuals)* | | |
|  |  | | |  | | |  | | |
| **Prof. attend (MBS)** |  | | |  | | |  | | |
| MCI | 0.003 | 0.043 | 0.946 | 0.017 | 0.045 | 0.695 | 0.007 | 0.045 | 0.879 |
| Dementia | **0.182^**^** | **0.076** | **0.017** | **0.225^***^** | **0.079** | **0.004** | 0.206 | 0.145 | 0.155 |
| *N (individuals)* | *1,609 (697 individuals)* | | | *1,502 (606 individuals)* | | | *1,531 (662 individuals)* | | |
|  |  | | |  | | |  | | |
| **Diag. imag. MBS)** |  | | |  | | |  | | |
| MCI | -0.117 | 0.075 | 0.119 | -0.097 | 0.078 | 0.212 | -0.127 | 0.080 | 0.111 |
| Dementia | -0.200 | 0.180 | 0.269 | -0.157 | 0.184 | 0.394 | -0.206 | 0.178 | 0.245 |
| *N (individuals)* | *1,324 (645 individuals)* | | | *1,236 (568 individuals)* | | | *1,259 (613 individuals)* | | |
|  |  | | |  | | |  | | |
| **Pathology (MBS)** |  | | |  | | |  | | |
| MCI | 0.014 | 0.072 | 0.841 | 0.017 | 0.076 | 0.822 | 0.002 | 0.086 | 0.980 |
| Dementia | -0.074 | 0.156 | 0.636 | -0.010 | 0.161 | 0.950 | -0.052 | 0.218 | 0.812 |
| *N (individuals)* | *1,450 (674 individuals)* | | | *1,350 (589 individuals)* | | | *1,380 (642 individuals)* | | |
|  |  | | |  | | |  | | |
| **All PBS** |  | | |  | | |  | | |
| MCI | -0.022 | 0.070 | 0.752 | 0.061 | 0.077 | 0.431 | -0.019 | 0.083 | 0.816 |
| Dementia | **0.888^**^** | **0.092** | **0.000** | **0.659^***^** | **0.119** | **0.000** | **0.889^***^** | **0.329** | **0.007** |
| *N (individuals)* | *2,265 (835 individuals)* | | | *1,962 (630 individuals)* | | | *2,185 (799 individuals)* | | |
|  |  | | |  | | |  | | |
| **Nerv. syst. (PBS)** |  | | |  | | |  | | |
| MCI | **0.302^**^** | **0.132** | **0.022** | **0.430^***^** | **0.162** | **0.008** | 0.275 | 0.183 | 0.132 |
| Dementia | **1.605^***^** | **0.131** | **0.000** | **2.084^***^** | **0.168** | **0.000** | **1.614^***^** | **0.204** | **0.000** |
| *N (individuals)* | *1,153 (551 individuals)* | | | *979 (422 individuals)* | | | *1,153 (551 individuals)* | | |
|  |  | | |  | | |  | | |

* Full modelling results for all covariates available from the authors. **p<0.1*, p<0.05**, p<0.01*****

**Table B4: Robustness check for part one model results – Marginal effects - percentage point change in probability of health care use associated with principal components analysis-computed z-score**

|  | ***(A1) Unbalanced sample***  ***(random effects logit)*** | | | ***(A3) IPW-weighted***  ***(pooled logit)*** | | |
| --- | --- | --- | --- | --- | --- | --- |
|  | ***Effect*** | ***S.E*** | ***P*** | ***Effect*** | ***S.E*** | ***P*** |
| **1 S.D ↑ in z-score:** |  |  |  |  | | |
| All MBS | **0.029^***^** | **0.007** | **0.000** | **0.023^***^** | **0.008** | **0.004** |
| *N (individuals)* | *2,690 (956 individuals)* | | | *2,606 (918 individuals)* | | |
|  |  | | |  | | |
| Prof. attend. (MBS) | **0.040^***^** | **0.011** | **0.000** | **0.038^***^** | **0.010** | **0.000** |
| *N (individuals)* | *2,690 (956 individuals)* | | | *2,606 (918 individuals)* | | |
|  |  |  |  |  | | |
| Diag. imaging (MBS) | **0.032^***^** | **0.012** | **0.006** | **0.029^***^** | **0.011** | **0.007** |
| *N (individuals)* | *2,690 (956 individuals)* | | | *2,606 (918 individuals)* | | |
|  |  |  |  |  | | |
| Pathology (MBS) | **0.042^***^** | **0.011** | **0.000** | **0.037^***^** | **0.010** | **0.000** |
| *N (individuals)* | *2,690 (956 individuals)* | | | *2,606 (918 individuals)* | | |
|  |  |  |  |  | | |
| All PBS | **0.030^***^** | **0.009** | **0.001** | **0.020^***^** | **0.009** | **0.019** |
| *N (individuals)* | *2,690 (956 individuals)* | | | *2,606 (918 individuals)* | | |
|  |  |  |  |  | | |
| Nerv. syst. drugs (PBS) | **-0.031^**^** | **0.014** | **0.021** | **-0.034^***^** | **0.011** | **0.003** |
| *N (individuals)* | *2,690 (956 individuals)* | | | *2,606 (918 individuals)* | | |

**Table B5: Robustness check for part two model results – Marginal effects - $ change in costs incurred for those who incurred any costs associated with principal components analysis-computed z-score**

|  | ***(C1) Unbalanced sample GEE*** | | | ***(C3) IPW-weighted GEE*** | | |
| --- | --- | --- | --- | --- | --- | --- |
|  | ***Effect*** | ***S.E*** | ***P*** | ***Effect*** | ***S.E*** | ***P*** |
| **1 S.D ↑ in z-score:** |  |  |  |  | | |
| All MBS | -177.475 | 120.512 | 0.141 | -151.497 | 141.597 | 0.285 |
| *N (individuals)* | *2,329 (835 individuals)* | | | *2,254 (800 individuals)* | | |
|  |  | | |  | | |
| Prof. attend. (MBS) | **-131.460^***^** | **42.773** | **0.002** | **-134.126^**^** | **52.120** | **0.010** |
| *N (individuals)* | *1,596 (680 individuals)* | | | *1,522 (645 individuals)* | | |
|  |  |  |  |  | | |
| Diag. imaging (MBS) | 56.544 | 47.282 | 0.232 | 70.199 | 65.195 | 0.282 |
| *N (individuals)* | *1,316 (635 individuals)* | | | *1,253 (602 individuals)* | | |
|  |  |  |  |  | | |
| Pathology (MBS) | **-48.658^**^** | **19.707** | **0.014** | **-42.328^**^** | **20.567** | **0.040** |
| *N (individuals)* | *1,434 (653 individuals)* | | | *1,368 (621 individuals)* | | |
|  |  |  |  |  | | |
| All PBS | **-334.009^***^** | **91.815** | **0.000** | **-337.912^**^** | **158.837** | **0.033** |
| *N (individuals)* | *2,284 (834 individuals)* | | | *2,209 (800 individuals)* | | |
|  |  |  |  |  | | |
| Nerv. syst. drugs (PBS) | **-62.548^***^** | **15.173** | **0.000** | **-59.880^**^** | **30.025** | **0.046** |
| *N (individuals)* | *1,139 (549 individuals)* | | | *1,097 (527 individuals)* | | |

**Table B6: Sample sizes for MCI subgroup (pooled waves 1-4)**

| ***Characteristic*** | ***Sample size (observations)*** |
| --- | --- |
| MCI and male | 488 |
| MCI and female | 494 |
|  |  |
| MCI and Managerial/Professional past occupation | 505 |
| MCI and Managerial/Professional past occupation | 477 |
|  |  |
| MCI with amnestic symptoms | 556 |
| MCI with no amnestic symptoms | 426 |
|  |  |
| MCI and lives alone | 451 |
| MCI and does not live alone | 533 |
|  |  |
| MCI and carer helps with making medical decisions | 277 |
| MCI and carer does not help with making medical decisions | 707 |
|  |  |
| MCI and carer helps with transport | 326 |
| MCI and carer does not help with transport | 658 |
|  |  |

**Table B7: Covariates mapped to Andersen’s Expanded Behavioural Model of health services use**

| ***Category in Andersen’s Model*** | ***Covariates*** |
| --- | --- |
|  |  |
| **Predisposing** | Age, gender, non-English speaking background, years of education |
|  |  |
| **Enabling** | Past occupation, marital status |
|  |  |
| **Need** | Number of diagnosed comorbidities, health-related quality of life |
|  |  |
| **Personal health practices** | Smoking status, alcohol consumption, physical activity participation |
|  |  |

Source: Andersen RM. ‘Revisiting the behavioral model and access to medical care: does it matter? Journal of health and social behavior’, 1995:1-10.
